# A Dinitrogen Complex without Donor Ligands: Isolation and Characterization of a [Mn(CO)_5_(η^1^‑N_2_)]^+^ Salt

**DOI:** 10.1021/jacs.5c13214

**Published:** 2025-09-13

**Authors:** Malte Sellin, James D. Watson, Julia Fischer, Manuel Schmitt, Graham E. Ball, Leslie D. Field, Ingo Krossing

**Affiliations:** † Institut für Anorganische und Analytische Chemie and Freiburg Materials Research Center FMF, 9174 Albert-Ludwigs-Universität Freiburg, Albertstr. 21, 79104 Freiburg, Germany; ‡ School of Chemistry, 7800 University of New South Wales, Sydney, Sydney, NSW 2052, Australia; § Anorganisch-Chemisches Institut Universität Heidelberg, Im Neuenheimer Feld 270, 69120 Heidelberg, Germany

## Abstract

Dinitrogen complexes are intermediates in nitrogen fixation. Until now, all isolated molecular dinitrogen complexes have relied on ancillary ligands that are net electron donors, yielding N_2_ ligands carrying a clear negative partial charge. Here, we present the synthesis, isolation, and characterization of the complex salt [Mn­(CO)_5_(η^1^-N_2_)]^+^[F­(Al­(OR^F^)_3_)_2_]^−^ (R^F^ = C­(CF_3_)_3_) that resulted from the oxidation of Mn_2_(CO)_10_ under a dinitrogen atmosphere in pentafluorobenzene solvent. The IR/Raman spectra reveal a high N_2_ frequency of 2301/2303 cm^–1^ close to free N_2_ gas (2330 cm^–1^) that indicates little π-back-donation. QTAIM and CD-NOCV analyses show that the carbonyl ligands act as net acceptor ligands that induce the formation of an inversely polarized dinitrogen ligand with a +0.2 charge on the terminal atom that holds the potential to be susceptible to nucleophilic attack.

## Graphical abstract

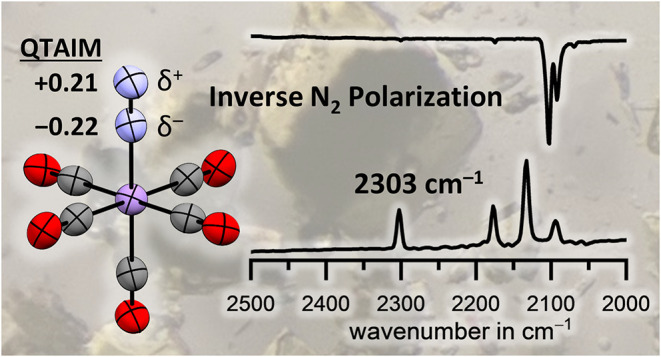

## Introduction

The activation of dinitrogen plays a pivotal role, both in industrial applications like the Haber-Bosch process^1–4^ and in biological systems.^5,6^ In particular, dinitrogen complexes featuring metals with a *d* ^6^ electron configuration have been extensively studied over the past six decades, as they serve as model systems for nitrogenases—enzymes that catalyze the conversion of dinitrogen to ammonia.^7–10^ With its poor σ-donor ability, the dinitrogen ligand primarily binds to the metal atom through π-back-donation. However, dinitrogen is also a relatively weak π-acceptor, and consequently, the formation of metal dinitrogen complexes typically requires electron-rich metal centers with strong σ-donor ligands such as amines or phosphines.^11,12^ Consequently, π-back-donation from the metal to the coordinated dinitrogen ligand weakens the N≡N bond in electron-rich dinitrogen complexes and generates a negatively polarized terminal β-nitrogen atom, facilitating reactions with electrophiles like protons. Hence, protonation of the terminal β-nitrogen is the initial step in the biological N_2_-activation and is a key feature of the Chatt and Schrock, as well as the newer modified Tuczek cycles.^13–15^ Thus, relative to free, gaseous N_2_ with *ν̃* (N_2_) at 2330 cm^–1^, the stretching frequency in such active complexes is typically red-shifted by up to 400 cm^–1^.^7–10,13–16^ In addition to their role as enzyme model systems, dinitrogen complexes are also utilized for the generation of ammonia by homogeneous catalysis.^17^ In particular, manganese dinitrogen complexes are gaining increasing attention in this field due to the earth abundance and low cost of manganese.^18–20^

Yet, the investigation of systems that activate dinitrogen in nonclassical ways is essential to develop new mechanisms for the conversion of dinitrogen into more valuable products. Here, we are interested in systems with an inverse (positive) polarization of the coordinated N_2_ molecule that may result from using very strong acceptor ligands and leave the *ν̃* (N_2_) stretching frequency at very high values, related to the nonclassical transition metal carbonyl cations.^21–24^ Known dinitrogen complexes with the highest stretching frequency *ν̃* (N_2_) are bulky trigonal planar copper­(I) imine complexes from Betley et al. (2242 cm^–1^),^25^ or the N_2_ adduct to an ion-paired copper­(I) perfluoroalkoxyaluminate from our group (2314 cm^–1^).^26^ However, both complexes are mainly used as reactive Cu­(I) binding sites with dinitrogen serving as a labile ligand, but not as an inversely polarized starting point for functionalization.^25,27^

However, systems with very strong acceptors have been studied in the gas phase via coupled mass spectrometry/IR spectroscopy or through matrix isolation techniques.^28–35^ Even homoleptic dinitrogen complexes M­(N_2_)_*x*_ and [M­(N_2_)_*x*_]^+^ are known (Figure 1). Similarly, the (photolytic) generation and investigation of mixed-ligand complexes with the general formula M­(CO)_*x*_(N_2_) has been limited to low-temperature environments (e.g., matrix isolation or liquid noble gases),^36,37^ gas-phase studies,^38^ or ultrafast pump–probe spectroscopy.^39^

**Figure 1:**
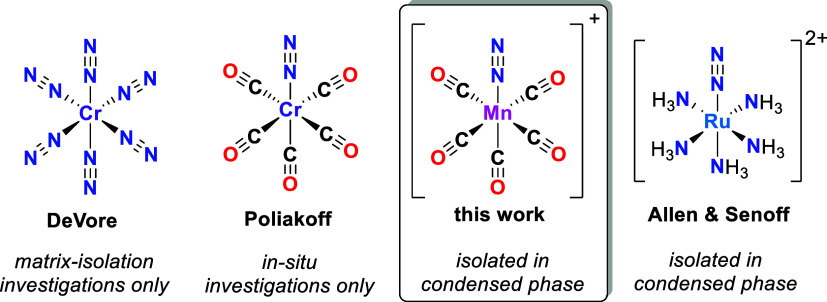
Currently known, isoelectronic *pseudo*-octahedral *d* ^6^ L_5_M­(N_2_) complexes and [Mn­(CO)_5_(N_2_)]^+^ (this work).

In conventional environments, these systems are prohibitively reactive, and there have yet to be any reports on the isolation of M­(CO)_*x*_(N_2_) complexes. As a further challenge, the back-reaction of such a photolytically generated complex with the liberated CO to give the homoleptic carbonyl complex is only kinetically hindered in closed systems and occurs readily already at 112 K for Ni­(CO)_3_(N_2_)^36^ and at 238 K for Cr­(CO)_5_(N_2_).^37^ Hence, the challenging synthesis of an acceptor-only mixed M­(CO)_*x*_(N_2_) complex as in Figure 1 is the target of this contribution.

### Suitable Precursors, Counterions, and Solvents

Coordinatively unsaturated isolable transition metal carbonyl complexes (TMCs) are unknown as potential precursors for N_2_ complexes without donor ligands—in contrast to other isoelectronic ligand classes, such as isocyanides.^40^ Approaching such unsaturated precursor states, Mews reported the isolation of a series of strongly Lewis-acidic cationic 16-valence electron (VE) complexes that were generated for manganese via halide (X^–^) abstraction from X-Mn­(CO)_5_ or by oxidation reactions from the Mn_2_(CO)_10_-dimer.^41^ Here, the products obtained were either sulfur dioxide adducts or ion pairs with the hexafluoroarsenate­(V) anion, for example, Mn­(CO)_5_(F–AsF_5_). To circumvent the formation of such ion pairs, even weaker coordinating anions (WCAs) than [AsF_6_]^−^ must be employed. The perfluorinated alkoxyaluminate WCAs [Al­(OR^F^)_4_]^−^ and [F­{Al­(OR^F^)_3_}_2_]^−^ (R^F^ = C­(CF_3_)_3_) developed by our group are among the least coordinating anions known and effectively remove the WCA as a limiting factor when stabilizing weakly bound ligands.^42,43^ Furthermore, since even the very weakly coordinating solvent sulfur dioxide competes with [AsF_6_]^−^ as a ligand and forms the salt [Mn­(CO)_5_(SO_2_)]^+^[AsF_6_]^−^, the need for weakly coordinating solvents may be addressed by using the highly fluorinated benzene derivatives 4FB (= 1,2,3,4-F_4_C_6_H_2_) or 5FB (= F_5_C_6_H).^44^ Both 4FB and 5FB are extremely weakly coordinating and are sufficiently polar to dissolve salts, at least when partnered with the aluminate WCAs described above. The last factor to consider when accessing such an N_2_ complex is the reagent used to generate the coordinatively unsaturated precursor fragment [Mn­(CO)_5_]^+^. We recently introduced a highly potent one-electron oxidant (or deelectronator),^45^ the perfluoronaphthalene radical cation,^46^ which is capable of oxidizing Mn_2_(CO)_10_ in the presence of *n*-pentane to form the corresponding alkane σ-complex [Mn­(CO)_5_(*n*-pentane)]^+^. The resultant neutral perfluoronaphthalene byproduct exhibits even less donor ability than *n*-pentane.^47^ Adopting this reaction strategy,^48^ we here report the synthesis, isolation, and characterization of the inversely polarized dinitrogen complex salt [Mn­(CO)_5_(η^1^-N_2_)]^+^[F­(Al­(OR^F^)_3_)_2_]^−^ ([**1**]^+^[F­{Al­(OR^F^)_3_}_2_]^−^) as a room-temperature stable crystalline solid.

## Results and Discussion

### Synthesis

When Mn_2_(CO)_10_ is reacted at −20 °C with [C_10_F_8_]^+•^[F­(Al­(OR^F^)_3_)_2_]^−^ in 4FB or 5FB solution and stirred for 5 min under nitrogen, the solution turned from the intense green of the naphthalene radical cation to almost colorless. After being warmed to room temperature, the solution was layered with *n*-pentane. Slow room-temperature diffusion over days yielded colorless to slightly yellow crystalline blocks suitable for single-crystal X-ray diffraction that were identified as the title compound [**1**]^+^[F­(Al­(OR^F^)_3_)_2_]^−^ with a 77% yield (eq 1). [**1**]^+^[F­(Al­(OR^F^)_3_)_2_]^−^ exhibits a minor N≡N stretch in the IR but an intense N≡N stretch in the Raman spectrum at 2301/2303 cm^–1^.
1

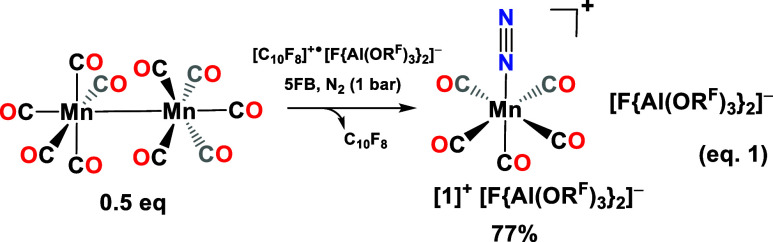




### Molecular Structure

The pale yellow to colorless crystalline blocks of the dinitrogen complex [**1**]^+^[F­(Al­(OR^F^)_3_)_2_]^−^ crystallize isostructurally to the homoleptic complex salt [Mn­(CO)_6_]^+^[F­(Al­(OR^F^)_3_)_2_]^−^ in cubic space group *P*a3̅, featuring only one crystallographically independent ligand. This crystallographic limitation, already known from previous reports on [Cr­(CO)_6–*x*_(NO)_*x*_]^+•^[F­(Al­(OR^F^)_3_)_2_]^−^ (*x* = 0, 1) salts,^49^ prevents a meaningful discussion of the structure of [**1**]^+^ based on single-crystal X-ray diffraction (scXRD) data alone, since the N_2_ ligands co-occupies the carbonyl position in a 1:5 ratio and one cannot differentiate between N_2_ and CO ligand based on scXRD only. Yet, the overall molecular structure as a (pseudo)­octahedral [Mn­(CO)_5_(η^1^-N_2_)]^+^ cation is proven together with the subsequent spectroscopic data.

### IR/Raman Spectroscopy

The vibrational IR/Raman spectra shown in Figure 2A feature a weak/strong band of the coordinated dinitrogen ligand at 2301/2303 cm^–1^ that provides conclusive evidence for dinitrogen coordination. Compared to free gaseous N_2_ (2330 cm^–1^), this band is red-shifted by less than ca. 30 cm^–1^.^16^ Only the recently reported copper complex [(η^1^-N_2_)­Cu­(Al­(OR^F^)_4_)] has a higher *ν̃* (N≡N) frequency of 2314 cm^–1^, while most other dinitrogen complexes display significantly by 100–400 cm^–1^ red-shifted N_2_ stretching vibrations.^7–10,13–15^ The carbonyl bands pattern of [**1**]^+^ is characteristic of the [Mn­(CO)_5_]^+^ fragment (Figure 2A and Table S2, Supporting Information (SI)) and closely resembles that of [Mn­(CO)_5_L]^+^ complexes (L = SO_2_, *n*-pentane)^41,47^ as well as neutral Mn­(CO)_5_X species (X = Br, OTeF_5_, F–AsF_5_).^41,50,51^ All observed frequencies are consistent with the calculated values (Figure 2A, B3LYP­(D3BJ)/def2-TZVPP). Notably, the ν­(CO) vibrations of [**1**]^+^ are blue-shifted by 10–20 cm^–1^ compared to those of [Mn­(CO)_5_(*n*-pentane)]^+^. This blue shift is expected since dinitrogen is a π-acceptor ligand that reduces the extent of π-back-donation to the carbonyl ligands. The complex still exhibits an average carbonyl stretching frequency (*ν̃* (CO)_av_) of 2122 cm^–1^ that is red-shifted compared to free gaseous carbon monoxide (2143 cm^–1^), supporting the classification of [Mn­(CO)_5_(η^1^-N_2_)]^+^ as a “classical carbonyl complex”.^52^ This red shift indicates that the carbonyl ligands carry partial negative charges and act as net acceptor, rather than net donor ligands.

**Figure 2:**
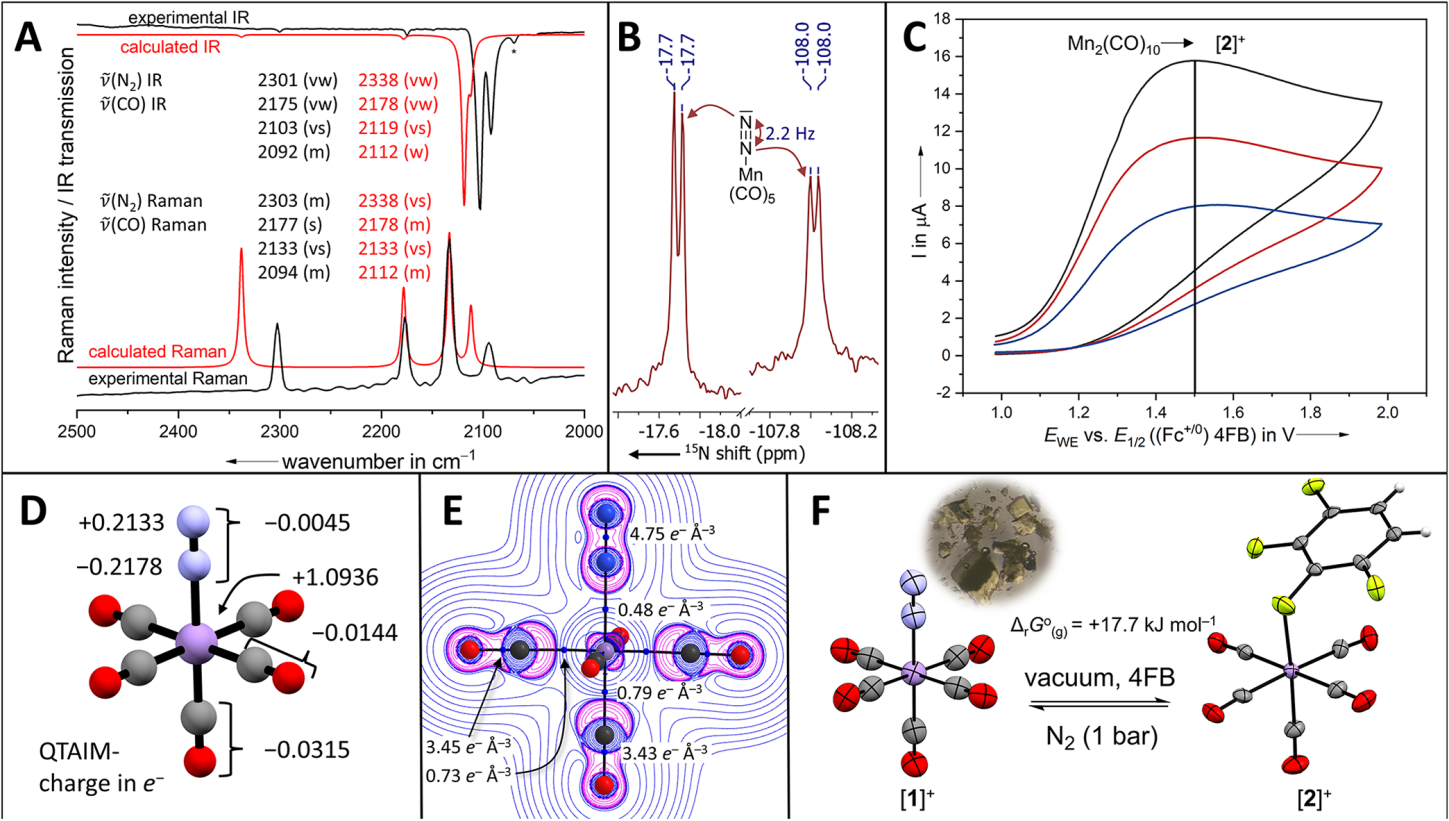
(A) Experimental (black lines) IR (top) and Raman (bottom) spectra of [**1**]^+^[F­(Al­(OR^F^)_3_)_2_]^−^ in comparison with the DFT-calculated vibrational spectra of [**1**]^+^ (red lines) at the B3LYP­(D3BJ)/def2-TZVPP level of theory scaled by 0.968 according to Duncan et al.^62^ (B) ^15^N NMR spectrum (51 MHz) of [Mn­(CO)_5_(η^1^-^15^N_2_)]^+^[F­(Al­(OR^F^)_3_)_2_]^−^ in HFP under a ^15^N_2_ atmosphere (1 bar) at −50 °C. (C) Cyclic voltammetry of Mn_2_(CO)_10_ (10 mM) in 4FB in an argon atmosphere with [NBu_4_]^+^[Al­(OR^F^)_4_]^−^ (100 mM) as supporting electrolyte. Scan rates: 50 (blue), 100 (red), and 200 mV s^–1^ (black). (D) Calculated QTAIM charges that reside on N atoms and CO ligands of [**1**]^+^ (B3LYP­(D3BJ)/def2-TZVPP). A list of the QTAIM charges of all atoms is deposited in Table S3 in the Supporting Information. (E) Laplacian of the electron density ∇^2^ρ­(*r*) in the N–Mn–C plane in [**1**]^+^ calculated at the B3LYP­(D3BJ)/def2-TZVPP level of theory; ∇^2^ρ­(*r*) > 0 in blue lines and ∇^2^ρ­(*r*) < 0 in pink lines; blue dots represent the bond critical points. (F) Conversion of [**1**]^+^ in [**2**]^+^. Δ*_r_G*_(g)_^o^ calc. at the DLPNO–CCSD­(T1)/def2-QZVPP//B3LYP­(D3BJ)/def2-TZVPP level of theory. Color code: manganese, purple; fluorine, light green; oxygen, red; nitrogen, light blue; carbon, gray; hydrogen, white. Displacement ellipsoids of molecular structures set at 50% probability. Disorder of the complex cations and counterions is not shown for clarity. Inset: single crystals of [**1**]^+^[F­(Al­(OR^F^)_3_)_2_]^−^ in perfluorinated oil.

### NMR Spectroscopy

The ^15^N NMR spectrum of [**1**]^+^ was obtained *in situ* by repeating eq 1 in 1,1,1,3,3,3-hexafluoropropane (HFP)[Fn fn1] at −50 °C to freeze out any inter- and intramolecular exchange reactions of the N_2_ unit. Using ^15^N-labeled dinitrogen, two sharp doublets were observed at −17.7 and −108.0 ppm (relative to MeNO_2_, Figure 2B). Given that ^15^N chemical shifts of dinitrogen ligands in transition metal complexes can span several hundred ppm and are highly metal-dependent,^53,54^ we computationally validated the assignments using DFT calculations at the PBE0/QZ4P level.^55,56^ The calculated shifts (−3.3/–105.5 ppm) are in excellent agreement with the experimental values. The magnitude of the ^1^ *J*_15N≡15N_ coupling constant in [**1**]^+^ is 2.2 Hz (Figure 2B), which is significantly smaller than couplings typically observed for other η^1^-coordinated dinitrogen complexes (4–7 Hz).^53,54^ This is likely indicative of a relatively weak interaction between the metal center and the dinitrogen ligand, given that the calculated ^1^ *J*_15N≡15N_ coupling constant in [**1**]^+^ of 1.74 Hz is almost identical to that calculated for free N_2_ (1.76 Hz, PBE0/QZ4P-J level).

### Cyclic Voltammetry

To elucidate the necessity of using the very strong oxidant [C_10_F_8_]^+•^[F­(Al­(OR^F^)_3_)_2_]^−^, we investigated the electrochemical oxidation of Mn_2_(CO)_10_ by cyclic voltammetry (CV) in 1,2,3,4-tetrafluorobenzene (4FB) under argon. The CV trace shown in Figure 2C displays an electrochemically irreversible oxidation event at +1.5 V vs Fc^+/0^, confirming the need for strong deelectronators to oxidize Mn_2_(CO)_10_. Note that mononuclear transition metal carbonyl complexes exhibit significantly lower and, by contrast, also reversible half-wave potentials, such as +1.21 V for Ni­(CO)_4_ and +0.86 V for Fe­(CO)_5_, which correspond both to oxidation potentials lower than +1.3/+1.0 V with the given scan rates (all vs Fc^+/0^ in 4FB).^57,58^

### Reaction with 4FB

When the dinitrogen atmosphere of the reaction solution is replaced by argon, the dinitrogen ligand in [**1**]^+^ is replaced by the 4FB solvent, leading—after slow diffusion with *n*-pentane—to the crystallization of the complex salt [Mn­(CO)_5_(η^1^–F-C_6_F_3_H_2_)]^+^[F­(Al­(OR^F^)_3_)_2_]^−^ ([**2**]^+^ = Mn­(CO)_5_(η^1^–F-C_6_F_3_H_2_)]^+^) (Figure 2F). In contrast to the few known 4FB complexes bound to rhodium,^59^ copper,^27^ and silver atoms,^44^ the 4FB ligand does not coordinate via the π-system of the benzene ring, but rather through a lone pair orbital of a fluorine atom. While this coordination mode is unprecedented for highly fluorinated arenes, it has been observed for less fluorinated analogues such as fluorobenzene and 1,2-difluorobenzene.^60,61^

### Computed Binding Energies

To validate the experimentally observed binding trends, we calculated a series of ligand-binding energies (Δ_r_ *E* and Δ_r_ *G*_(g)_^o^) to the [Mn­(CO)_5_]^+^ fragment at the DLPNO–CCSD­(T1)/def2-QZVPP//B3LYP­(D3BJ)/def2-TZVPP level of theory (Table 1). As expected, 4FB and *n*-pentane exhibit the weakest interactions, with binding energies Δ_r_ *E*/Δ_r_ *G*_(g)_^o^ of −92.7/–37.5 and −99.3/–44.3 kJ mol^–1^, respectively. Dinitrogen binds more strongly at −105.5/–55.2 kJ mol^–1^. However, when compared to stronger ligands such as carbon monoxide (−181.8/–122.1 kJ mol^–1^) and the strong σ-donor PMe_3_ (−303.6/–237.5 kJ mol^–1^), it is evident that all experimentally observed complexes display relatively weak ligand–metal interactions. Local energy decomposition (LED) analyses (conducted at the DLPNO–CCSD­(T1)/def2-QZVPP//B3LYP­(D3BJ)/def2-TZVPP level of theory) break down the binding interactions in these species and indicate that for the weaker-bound 4FB, pentane, and N_2_ complexes, the dispersion interaction *E*_disp._(LED) accounts for more than 40% of the overall binding energy. In the N_2_ complex [**1**]^+^, *E*_disp._(LED) between the ligand and the [Mn­(CO)_5_]^+^ fragment corresponds to 43% of the net electronic binding energy, compared to even 54% in the *n*-pentane complex (bound through C3). In complexes with stronger σ-donors such as PMe_3_, the dispersion contribution to the binding is considerably lower percentage (26%). SI Section 6.3 contains further information and dispersion interaction density (DID) plots.

**Table 1:** Calculated Binding Energies Δ*_r_E*_(g)_ and Δ*_r_G*_(g)_^o^ of [Mn­(CO)_5_]^+^ with Selected Ligands L to Give [(L)­Mn­(CO)_5_]^+^ in kJ mol^–1^ a

[(L)Mn(CO)_5_]^+^	Δ_r_ *E*_(g)_	Δ_r_ *G*_(g)_^o^	*E*_disp._(LED)/%^c^
L = PMe_3_	–303.6	–237.5	–78.6/26
L = CO	–181.8	–122.1	–70.9/39
L = SO_2_	–121.6	–69.8	–39.8/33
L = N_2_	–105.5	–55.2	–45.2/43
L = *n*-pentane^b^	–99.3	–44.3	–53.2/54
L = 4FB	–92.7	–37.5	–37.6/41

a The local energy decomposition (LED) analyses giving *E*_disp._(LED) between the ligand and the [Mn­(CO)_5_]^+^ fragment are given in kJ mol^–1^ and as % of the total interaction. All entries were calculated at the DLPNO–CCSD­(T1)/def2-QZVPP//B3LYP­(D3BJ)/def2-TZVPP level of theory. Δ_r_ *G*_(g)_^o^ is given at 298 K and 1 atm.

b *n*-pentane bound through the C3-atom.

c = (*E*_disp._(LED)/Δ_r_ *E*_(g)_) × 100.

### QTAIM Analysis

The QTAIM analysis validates the role of the carbonyl ligands as net acceptors and not as net donor ligands. Both the carbonyl ligands *cis*- and *trans*-positioned toward the dinitrogen ligand carry a partial negative charge. Since dinitrogen is a weaker π-acceptor ligand as carbon monoxide, the carbonyl ligand *trans* to the dinitrogen moiety is bound stronger than the carbonyl ligand *cis* to it—indicated by the higher electron density at the bond critical points in Figure 2E. This underlines the weaker *trans* influence of the N_2_ ligand in comparison to CO. Additionally, the QTAIM analysis yields a negligible charge of −0.0045 on the N_2_ moiety and a significantly positively polarized terminal β-nitrogen atom that bears an unusual positive partial charge of +0.2133 (Figure 2D). Therefore, the polarization of the terminal β-nitrogen atom is inverted compared to that of classical dinitrogen complexes. This inverted polarization could potentially open new pathways for dinitrogen activation using nucleophiles instead of electrophiles. Note that weak inverse polarizations have also been calculated for some iron­(II) dinitrogen complexes at the weak activation limit using natural population analysis (NPA).^63^

### Charge Displacement Analysis

To further shed light on the unique electronic situation of the dinitrogen ligand in [**1**]^+^, the interaction between the [Mn­(CO)_5_]^+^ fragment and the N_2_ ligand was studied using the charge displacement analysis based on natural orbitals of chemical valence (CD-NOCV)^64,65^ at the BLYP/TZ2P level of theory. The amount of charge transfer (CT) associated with σ-donation (CT_σ_) is 0.13 e (Figure 3A), which falls within the typical range observed for isolated dinitrogen complexes (0.07–0.20 e). In contrast, the charge transfer in the opposite direction arising from the π-back-donation (CT_π_) amounts to only −0.14 e (Figure 3A). To the best of our knowledge, this represents the lowest value calculated for any isolated dinitrogen complex examined to date. Typically, CT_π_ lies between −0.19 and −0.63. As a result, the net charge transfer (CT_net_) between the [Mn­(CO)_5_]^+^ fragment and the N_2_ ligand is minimal, amounting to only −0.02 (typically between −0.08 and −0.43), in line with the QTAIM analysis. Therefore, the interaction of the dinitrogen ligand in [**1**]^+^ more closely resembles that calculated for the elusive homoleptic transition metal dinitrogen complexes like [Mn­(N_2_)_6_]^+^ unknown as isolable compounds, rather than any previously isolated heteroleptic dinitrogen complexes (Figure 3B). The comparison of the difference between the deformation densities between [**1**]^+^ and the typical electron-rich dinitrogen complex Mo­(PMe_3_)_5_(η^1^-N_2_) visualizes the variance in their interactions (Figure 3C,D). Both the σ-donation and the π-back-donation interaction lead to a positive polarization of the terminal nitrogen atom in [**1**]^+^ (CT_net_^N_2_^, Figure 3A).

**Figure 3:**
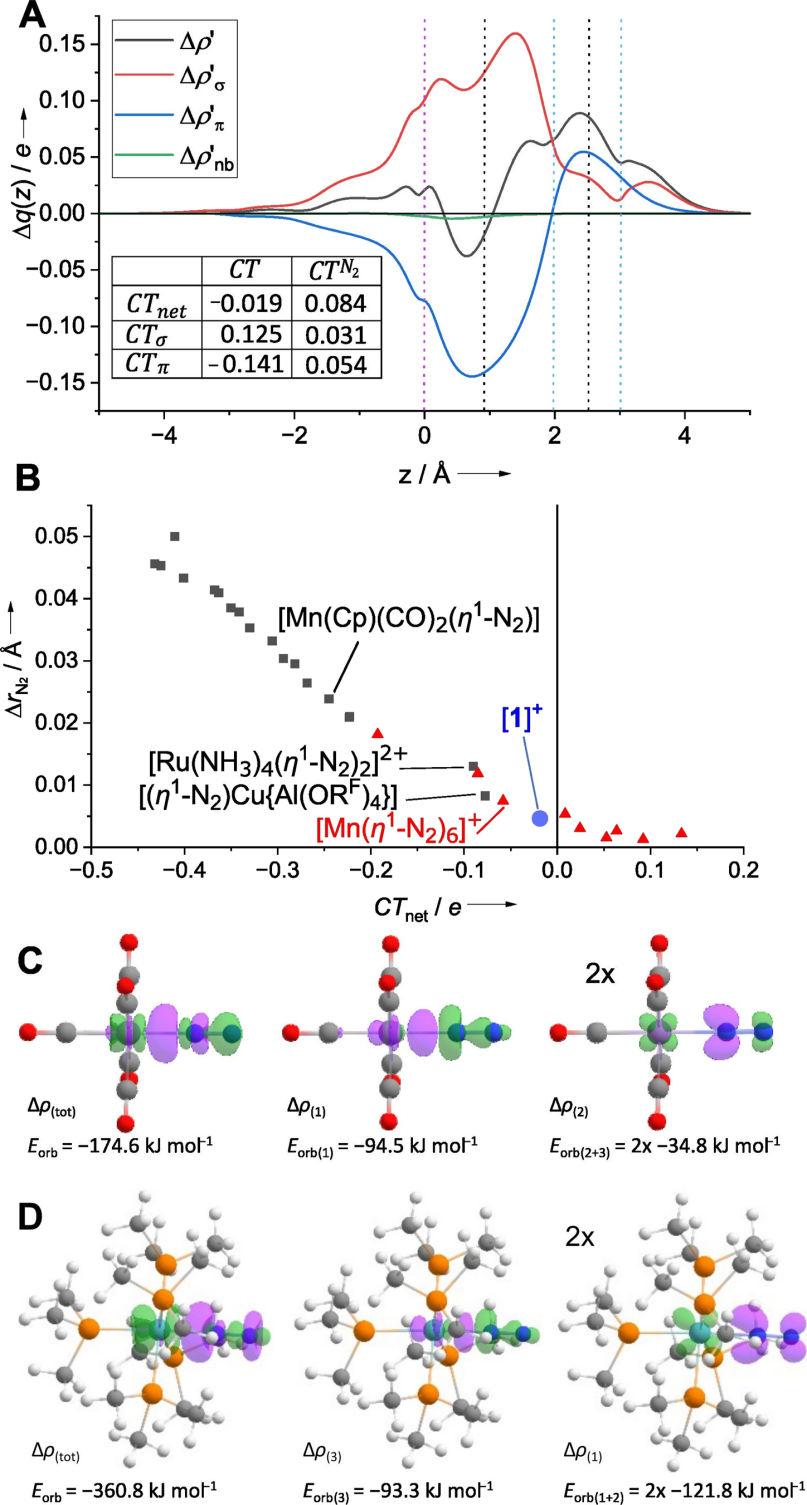
(A) Charge displacement functions of the σ-, π-, and nonbonding component of [**1**]^+^ together with their sum Δρ′. The dotted lines represent the positions of the atoms (purple, manganese; light blue, nitrogen) and the isodensity boundaries between the separated fragments on the *z*-axis (black). (B) Total charge transfer (CT_net_) from the CD-NOCV analysis was calculated against the change of the N_2_ distance relative to the calculated distance of free N_2_ (*r* = 1.1028 Å). Data for [(η^1^-N_2_)­Cu­(Al­(OR^F^)_4_)] taken from ref 26 other data except [**1**]^+^ taken from ref 64. Types of dinitrogen complexes are color coded: previously isolated dinitrogen complexes (black squares), homoleptic dinitrogen complexes from MS/matrix isolation work (red triangles), and [**1**]^+^ (blue spheres). (C, D) Shapes of the deformation densities (isodensity value 0.005 e B^–3^) upon the interaction of [Mn­(CO)_5_]^+^ (C) and [Mo­(PMe_3_)_5_] (D) with N_2_. Δρ′ (left), Δρ′_σ_ (middle), and Δρ′_π_ (right). Electron flow from green to purple.

## Conclusions

We report the synthesis and characterization of the dinitrogen complex [Mn­(CO)_5_(η^1^-N_2_)]^+^ devoid of net donor ligands, obtained via oxidation of Mn_2_(CO)_10_ under *pseudo gas-phase conditions* ^48^ in a dinitrogen atmosphere. The high N≡N stretching frequency observed in the Raman spectrum (2303 cm^–1^) indicates minimal π-back-bonding. Vibrational spectroscopy and QTAIM and CD-NOCV analyses show that the carbonyl ligands act as net acceptors, establishing this compound as the first isolated dinitrogen complex stabilized solely by acceptor ligands. With its strong π-acceptor character, the [Mn­(CO)_5_]^+^ fragment has almost no π-basicity, which overall results in negligible net charge transfer to the N_2_ ligand. In contrast, the terminal nitrogen atom even carries a significantly positive partial charge of +0.21 (QTAIM), representing the first example of a reasonably strongly bound dinitrogen complex with an inverted polarization. This inverted polarization in a reasonably stable complex could in the future potentially open new pathways for dinitrogen activation using weak and suitable nucleophiles instead of electrophiles. Investigations in this direction are underway but exceed the scope of this report.

## Supplementary Material



## Data Availability

The data that support the findings of this study are available in the Supporting Information of this article. Deposition numbers 2455464 (for [**1**]^+^[F­{Al­(OR^F^)_3_}_2_]^−^) and 2455463 (for [**2**]^+^[F­{Al­(OR^F^)_3_}_2_]^−^) accessible at https://www.ccdc.cam.ac.uk/services/structures contain the supplementary crystallographic data for this paper. These data are provided free of charge by the joint Cambridge Crystallographic Data Centre and Fachinformationszentrum Karlsruhe http://www.ccdc.cam.ac.uk/structures.

## Footnotes

1 HFP has a boiling point around 0 °C. Although J-Young NMR tubes can withstand the pressure of tubes filled with HFP at room temperature, it is recommended to handle them below the boiling point of HFP to avoid the possible risk of explosion.

## Notes

Contains synthetic procedures, additional spectra, and result of the DFT calculations in more detail (PDF)
