# Supplementary material for: A Dinitrogen Complex without Donor Ligands: Isolation and Characterization of a [Mn(CO)5(η1‑N2)]+ Salt
Source: J Am Chem Soc. 2025 Sep 13;147(38):35164–71. doi: 10.1021/jacs.5c13214 (PMC12464980; doi:10.1021/jacs.5c13214)
Supplement: Supplementary file 1 [file ja5c13214_si_001.pdf]

Supplementary Information

to

A Dinitrogen Complex without Donor-Ligands: Isolation and Characterization of a $[Mn(CO)_5(\eta^1\text{-}N_2)]^+$ Salt.

Authors: Malte Sellin,[1] James D. Watson,[2] Julia Fischer,[1] Manuel Schmitt,[3] Graham E. Ball,[2] Leslie D. Field,[2] Ingo Krossing[1*]

Affiliations:

[1] Dr. M. Sellin, B. Sc. J. Fischer, Prof. Dr. I. Krossing*
Institut für Anorganische und Analytische Chemie and Freiburg Materials Research Center FMF
Albert-Ludwigs-Universität Freiburg
Albertstr. 21, 79104 Freiburg (Germany)
E-mail: krossing@uni-freiburg.de

[2] Dr. J. D. Watson, Prof. Dr. G. E. Ball, Prof. Dr. L. D. Field
School of Chemistry
University of New South Wales, Sydney
Sydney, NSW 2052, Australia

[3] Dr. M. Schmitt
Anorganisch-Chemisches Institut
Universität Heidelberg
Im Neuenheimer Feld 270, 69120 Heidelberg (Germany)

Contents

1. General Procedures 2
2. Synthetic Procedures 5
3. Single Crystal X-Ray Diffraction 6
4. Vibrational Spectroscopy 9
5. NMR Spectroscopy 11
6. DFT Calculations 12
   6.1 AIM Analysis 12
   6.2 NMR Calculations 13
   6.3 Dispersion Interaction Density (DID) Plots 14
   6.4 DFT Optimized Structures 15

Summary of the calculated binding energies for the $Mn(CO)_5^+$ complexes 20
7 References...21

1. General Procedures

All manipulations were carried out by using standard Schlenk technique or a nitrogen filled glovebox (O_2/H_2O < 0.1 ppm). All the reactions were performed in Schlenk tubes with grease free PTFE-valves. The solvent 1,2,3,4-tetrafluorobenzene (4FB, $C_6F_4H_2$, from Fluorochem) was stirred a few days over calcium hydride (CaH_2) and distilled. The distillate was stirred over $Ag^+[Al(OR^F)_4]^-$ and condensed to remove traces of less fluorinated benzenes. This leads to a minor contamination of R^FOH (<<1 %), which does not affect the reactions. The solvent pentafluorobenzene (5FB) was stirred over calcium hydride (CaH_2) for 48 hours and distilled. *n*-Pentane was dried using a Grubbs apparatus. 4FB, 5FB, HFP and *n*-pentane were stored over 3 Å molar sieves. Octafluoronaphthalene (ABCR) and $Mn_2(CO)_{10}$ (ChemPur) were bought from commercial sources and used as received. $[C_{10}F_8]^{+\cdot}[F\{Al(OR^F)_3\}_2]^-$ and $Ag^+[Al(OR^F)_4]^-$ were prepared according to literature procedures.[1,2] $^{15}N_2$ 98% was purchased from Sigma-Aldrich and used without further purification.

<u>Vibrational Spectroscopy</u>

FTIR spectra were recorded inside a glovebox with a Bruker ALPHA equipped with QuickSnap Eco ATR module and ZnSe crystal. The spectra were measured at RT in the range of 4000−550 cm^{-1} (ZnSe) with 32 scans and a resolution of 2 cm^{-1}. The data were processed with the Bruker OPUS 7.5 software package. FT Raman spectra were recorded with a VERTEX 70 with Bruker RAM II Modul (1064 nm exciting line of a Nd-YAG laser) and liquid nitrogen cooled Ge detector. The samples were flame-sealed in soda-lime glass Pasteur pipettes and were measured at RT in the range of 4000−30 cm^{-1} with 1,000 scans and a resolution of 4 cm^{-1} with a laser power of 25 mW. The intensities are reported as follows: ≥ 0.8 = very strong (vs), ≥ 0.6 = strong (s), ≥ 0.4 = medium (m), ≥ 0.2 = weak (w), < 0.2 = very weak (vw). The data were processed with the Bruker OPUS 7.5 software package. The graphical representations were created with ORIGINPRO 2021b.

<u>NMR Spectroscopy</u>

NMR spectra were recorded at 222 K on a Bruker Advance NEO equipped with a TBI triple resonance probe operating at 500 MHz and 50 MHz for ^{1}H and ^{15}N nuclei respectively. ^{1}H NMR spectra were calibrated by setting the resonance of the solvent 1,1,1,3,3,3-hexafluoropropane to δ = 2.91 ppm, rel. to tetramethylsilane. ^{15}N NMR spectra were calibrated relative to nitromethane using the absolute referencing method using a value of Ξ ^{15}N = 10.136767. MestReNova and TopSpin software packages were used for processing and creating the graphical representations of the spectra.

<u>Preparation of NMR Samples</u>

Precautionary safety note: 1,1,1,3,3,3-Hexafluoropropane (HFP) boils at ca. 272 K: Samples must be kept at temperatures well below 270 K to mitigate the risk of explosion by the build-up of pressure if $CF_3CH_2CF_3$ is used above its boiling point in a sealed vessel.

<u>Sample Preparation for *in situ* NMR Experiment</u>

Typically, a pre-dried J. Young NMR tube and standard J. Young NMR tube piston, were cycled into an argon filled glove box. 1 mg of dimanganese decacarbonyl and 15 mg (2.0 eq.) of the deelectronator salt, $[C_{10}F_8]^{+\cdot}[F\{Al(OR^F)_3\}_2]^-$ were charged into the bottom of the tube before the tube was sealed using the J. Young NMR tube piston. Samples were cycled out of the glove box before being dried *in vacuo*

to remove any volatile contaminants. After the complex had been exposed to a vacuum, HFP (600-700 µL) was transferred onto the reagents *via* trap-to-trap vacuum distillation. Samples were mixed using a vortex at mixer −40 °C until homogenous. While mixing, the samples typically changed color from dark green to pale green/yellow. Once homogeneous, the samples were frozen once more and an atmosphere of ^{15}N isotope labelled dinitrogen was put onto the J. Young NMR tube. Once the $^{15}N_2$ was added to the reaction mixture, the samples were mixed briefly before being stored in an ethanol cold bath at temperatures below −40 °C and transported to the NMR spectrometer ready for analysis. Samples were removed from the cold bath and wiped several times with dry tissue paper to remove excess ethanol prior to inserting into a precooled and shimmed NMR spectrometer.

Single Crystal X-ray Diffraction

The data were collected on a Bruker D8 VENTURE dual wavelength Mo/Cu three-circle diffractometer with a microfocus sealed X-ray tube using mirror optics as monochromator and a Bruker PHOTON III detector. Single crystals were selected at RT in PFPE oil JC 1800 (Sunoit Performance Material Science), mounted on CryoLoops with a diameter of 0.1 to 0.2 mm and shock-cooled using an Oxford Cryostream 800 low temperature device. The data were gathered at 100(2) K using Mo K_α radiation (λ = 0.71073 Å). All data were integrated with SAINT (version 8.38A) and a multi-scan absorption correction using SADABS or TWINABS was applied. The structures were solved by direct methods using SHELXT[3] and refined by full-matrix least-squares methods against F^2 by SHELXL-2018/3[4] using the GUI software ShelXle.[5] Disordered moieties were refined using bond lengths restraints and displacement parameter restraints and were modelled with the program DSR.[6,7] The gathered data were finalized with the tool FinalCif.[8] The graphical representations of the crystal structures were generated with Mercury (version 4.0).[9] Crystallographic data for the structures reported in this paper have been deposited with the Cambridge Crystallographic Data Centre.[10] Copies of the data can be obtained free of charge from the Cambridge Crystallographic Data Centre via www.ccdc.cam.ac.uk/structures.

Cyclic Voltammetry

The cyclic voltammograms were recorded in an argon filled glovebox (O_2/H_2O < 0.1 ppm). A three-electrode arrangement was used with a 1 mm diameter platinum disc working electrode, a platinum mesh as counter electrode and a platinum wire in a compartment as a reference. The reference electrode is placed in a glass compartment with a frit to allow a direct measurement against a Pt|$Fc^{+/0}$ reference electrode.[11]

The solution in the compartment electrode has a mixture of 100 mM $[NBu_4]^+[Al(OR^F)_4]^-$, 10 mM Fc and 10 mM $[Fc]^{+\cdot}[Al(OR^F)_4]^-$ in 4FB. The analyte solution is composed from 100 mM $[NBu_4]^+[Al(OR^F)_4]^-$ and 10 mM analyte in 4FB. A VMP3 potentiostat (Bio-LogicScience Instruments) was used for the measurements, controlled *via* PC using the software EC-Lab (V11.21). The graphical representations were created with OriginPro 2021.

Computational Details

Geometry optimisations were performed with the TURBOMOLE software[12,13] (v7.2 or v7.5) using the DFT functionals B3LYP[14,15] with the def2-TZVPP[16] basis set, the resolution-of-identity (RI) approximation,[17–19] dispersion correction (D3BJ),[20] a fine integration grid (m4) and the default SCF convergence criteria (10^{-6} a.u.). All structures were checked for proper spin occupancies and imaginary frequencies with the integrated *EIGER* and *AOFORCE*[21] modules. The vibrational spectra were simulated at B3LYP(D3BJ)/def2-TZVPP level with a scaling factor of 0.968.[22] QTAIM-charges were calculated with MultiWFN.[23] The calculation of the bond critical points and the visualization of the relief map of the Laplacian of the electron density were performed with AIMAll. The charge displacement analysis based on natural orbitals of chemical valence (CD-NOCV) was performed as

described in the literature.[24] The BLYP functional,[15,25] a Slater type all electron triple-zeta basis set with two polarization functions (TZ2P)[26] and the D3BJ dispersion correction[20] were used. Relativistic effects were taken into account with the zeroth-order regular approximation (ZORA).[27–29]

Single point energies and LED calculations were performed at the DLPNO-CCSD(T1)/def2-QZVPP// B3LYP(D3BJ)/def2-TZVPP level of theory calculated using ORCA (version 6.0.1) software, tightPNO keyword throughout and using the iterative procedure for improved triples correction.[30]

NMR chemical shifts and coupling constants were calculated using the ADF software package v.2024.101 with the B3LYP(D3BJ)/def2-TZVPP geometries. Spin–orbit level relativity treatment was used, and the numerical quality was set to very good. All calculations used the PBE0 functional.[31] For calculation of the chemical shifts, the all-electron basis set QZ4P was used. The ^{15}N chemical shifts are calculated by subtracting the calculated shielding from the shielding of nitromethane calculated at the same level of theory without further scaling. Coupling constants were calculated, with the inclusion of all contributing terms, using the QZ4P-J basis set. A Gaussian finite nuclear model was used.

DID plots were generated using ChimeraX, developed by the Resource for Biocomputing, Visualization, and Informatics at the University of California, San Francisco, with support from National Institutes of Health R01-GM129325 and the Office of Cyber Infrastructure and Computational Biology, National Institute of Allergy and Infectious Diseases.

2. Synthetic Procedures

$[Mn(CO)_5(\eta^1\text{-}N_2)]^+[F\{Al(OR^F)_3\}_2]^-$ ($[\mathbf{1}]^+[F\{Al(OR^F)_3\}_2]^-$)

$[C_{10}F_8]^{+\cdot}[F\{Al(OR^F)_3\}_2]^-$ (46 mg, 26 µmol, 2.0 eq.) and $Mn_2(CO)_{10}$ (4.8 mg, 13 µmol, 1.0 eq.) were dissolved in 5FB or 4FB (2 mL) at −20 °C and stirred for five minutes under nitrogen. The solution turned from an intense green to almost colorless. Afterwards, the solution was layered with *n*-pentane (10 mL). Slow diffusion over days yielded colorless to yellow crystalline blocks suitable for single-crystal X-ray diffraction. The solvent mixture was removed and the crystals were washed with *n*-pentane multiple times to obtain pure $[\mathbf{1}]^+[F\{Al(OR^F)_3\}_2]^-$ (34 mg, 20 µmol, 77 %).

ATR-IR (ZnSe, microcrystalline powder) $\tilde{\nu}$ / cm^{-1} = 2301 (vw), 2175 (vw), 2103 (s), 2092 (m), 2070 (vw), 1533 (vw), 1515 (vw), 1356 (vw), 1301 (w), 1278 (m), 1249 (vs), 1218 (vs), 1186 (m), 1129 (vw), 1074 (vw), 974 (vs), 865 (vw), 840 (vw), 760 (vw), 727 (vs), 641 (w), 619 (m), 575 (vw), 569 (vw).

Raman $\tilde{\nu}$ / cm^{-1} = 2303 (m), 2177 (s), 2133 (vs), 2094 (m), 816 (m), 753 (m), 719 (m), 625 (m), 579 (m), 539 (m), 471 (m), 438 (m), 426 (m), 408 (m), 365 (m), 327 (m), 293 (m), 232 (m), 119 (vs).

^{15}N NMR (51 MHz, 1,1,1,3,3,3-Hexafluoropropane) δ = –17.7 (d, 1N, N^α, $^1J_{N\text{-}N}$ = 2.2 Hz), –71.2 (s, dissolved N_2), –108.0 (d, 1N, N^β, $^1J_{N\text{-}N}$ = 2.2 Hz) ppm.

^1H NMR (500 MHz, 1,1,1,3,3,3-Hexafluoropropane) Note: the compound contains no ^1H nuclei – spectrum shows impurities and solvent only. δ = 3.74 (quar., chlorotrifluoroethane impurity from solvent), 2.91 (hept., HFP solvent), 0.65, 0.43, 0.41, 0.37, –1.13, –1.31, –1.69 (minor unknown impurities) ppm.

$[Mn(CO)_5(\eta^1\text{-}F_4C_6H_2)]^+[F\{Al(OR^F)_3\}_2]^-$ ($[\mathbf{2}]^+[F\{Al(OR^F)_3\}_2]^-$)

$[C_{10}F_8]^{+\cdot}[F\{Al(OR^F)_3\}_2]^-$ (46 mg, 26 µmol, 2.0 eq.) and $Mn_2(CO)_{10}$ (4.8 mg, 13 µmol, 1.0 eq.) were dissolved in 4FB (2 mL) at −20 °C and stirred for a few minutes under nitrogen. The solution turned from an intense green to almost colorless. Subsequently, the solution was freeze-pumped-thawed two times and the headspace was refilled with argon gas. Afterwards, the solution was layered with *n*-pentane (10 mL). Slow diffusion over days yielded colorless to yellow crystalline blocks suitable for single-crystal X-Ray diffraction. The product was not further analyzed and no yield was determined.

3. Single Crystal X-Ray Diffraction

[**1**]$^+$[F{Al(OR^F)$_3$}$_2$]$^-$ features only one crystallographically independent ligand at the manganese atom, this issue was modelled with a mixed occupation (⅙ N_2; ⅚ CO). From the spectroscopic characterization (IR, Raman, NMR), other complexes for the type [Mn(CO)$_{6-x}$(η^1-N$_2$)$_x$]$^+$ with x ≠ 1 could be excluded. While a disorder of the [**1**]$^+$ in the Part –1 would have been possible, this was not meaningful, as carbon, nitrogen and oxygen have similar scattering factors and this would model six atoms at the same spot, resulting in a strong overparameterisation.

Table S1: scXRD data of [**1**]$^+$[F{Al(OR^F)$_3$}$_2$]$^-$ and [**2**]$^+$[F{Al(OR^F)$_3$}$_2$]$^-$.

Compound	[**1**]$^+$[F{Al(OR^F)$_3$}$_2$]$^-$	[**2**]$^+$[F{Al(OR^F)$_3$}$_2$]$^-$
CCDC number	2455464	2455463
Empirical formula	$C_{29}Al_2F_{55}MnN_2O_{11}$	$C_{35}H_2Al_2F_{59}MnO_{11}$
Formula weight	1706.21	1828.27
Temperature [K]	100(2)	100(2)
Crystal system	cubic	triclinic
Space group (number)	$Pa\overline{3}$ (205)	$P\overline{1}$ (2)
a [Å]	17.272(8)	10.562(9)
b [Å]	17.272(8)	12.659(12)
c [Å]	17.272(8)	21.078(18)
α [°]	90	83.63(3)
β [°]	90	79.16(3)
γ [°]	90	84.43(3)
Volume [Å^3]	5153(7)	2742(4)
Z	4	2
ρ_{calc} [gcm^{-3}]	2.199	2.214
μ [mm^{-1}]	0.547	0.531
F(000)	3288	1764
Crystal size [mm^3]	0.164×0.179×0.213	0.114×0.149×0.163
Crystal colour	yellow	colourless
Crystal shape	block	block
Radiation	MoK_α (λ=0.71073 Å)	MoK_α (λ=0.71073 Å)
2θ range [°]	4.08 to 61.08 (0.70 Å)	1.98 to 58.48 (0.73 Å)
Index ranges	−24 ≤ h ≤ 14<br>−17 ≤ k ≤ 23<br>−24 ≤ l ≤ 24	−14 ≤ h ≤ 14<br>−17 ≤ k ≤ 17<br>−28 ≤ l ≤ 28
Reflections collected	40694	56043
Independent reflections	2636<br>R_{int} = 0.0352<br>R_{sigma} = 0.0152	14898<br>R_{int} = 0.0316<br>R_{sigma} = 0.0309
Completeness to<br>θ = 25.242°	100.0 %	100.0 %
Data / Restraints / Parameters	2636 / 967 / 279	14898 / 8946 / 1188
Absorption correction T_{min}/T_{max}<br>(method)	0.7114 / 0.7461<br>(multi-scan)	0.6742 / 0.7458<br>(multi-scan)
Goodness-of-fit on F^2	1.105	1.017
Final *R* indexes<br>[I≥2σ(I)]	R_1 = 0.0359<br>wR_2 = 0.0772	R_1 = 0.0453<br>wR_2 = 0.1106
Final *R* indexes<br>[all data]	R_1 = 0.0494<br>wR_2 = 0.0827	R_1 = 0.0631<br>wR_2 = 0.1217
Largest peak/hole [eÅ^{-3}]	0.27/−0.23	1.32/−1.56

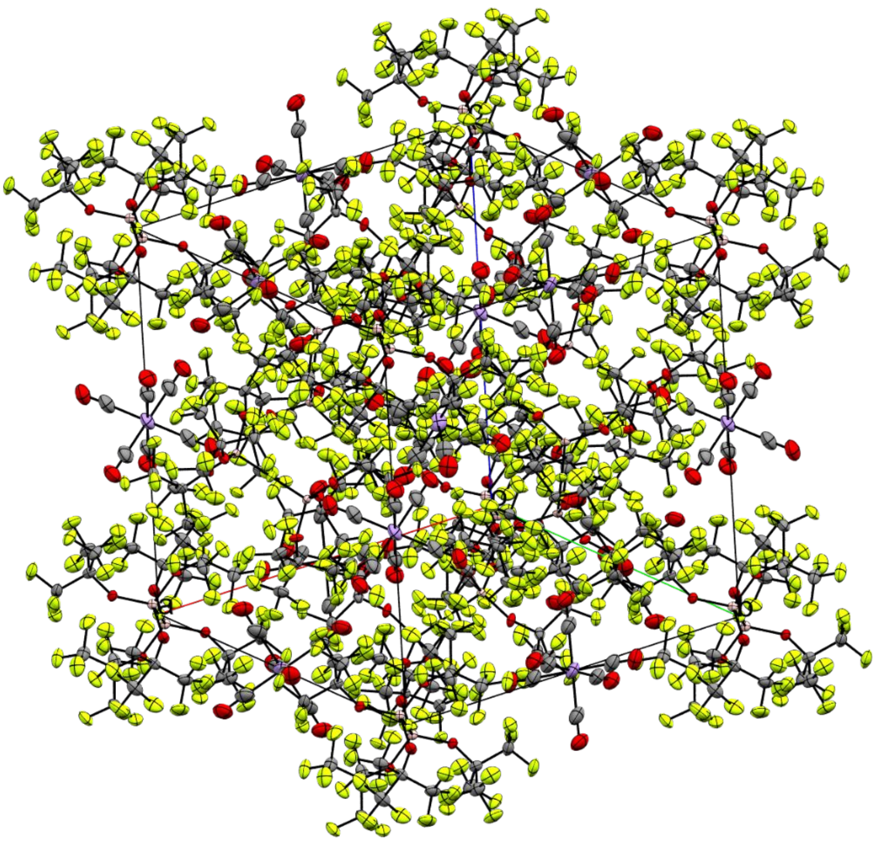

Figure S1: Unit cell of [**1**]$^+$[F{Al(OR^F)$_3$}$_2$]$^-$. Displacement ellipsoids shown at 50 % probability level. Color code: manganese – lavender, aluminium – rose, fluorine – light green, oxygen – red, carbon – grey, hydrogen – white, the mixed CO/N_2 positions are displayed as CO.

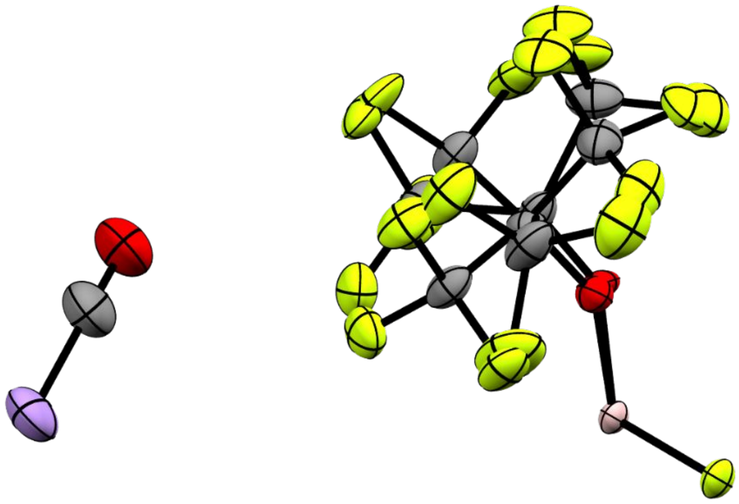

Figure S2: Asymmetric unit of [**1**]$^+$[F{Al(OR^F)$_3$}$_2$]$^-$ with anion disorders. Displacement ellipsoids shown at 50 % probability level. Color code: manganese – lavender, aluminium – rose, fluorine – light green, oxygen – red, carbon – grey, hydrogen – white, the mixed CO/N_2 positions are displayed as CO.

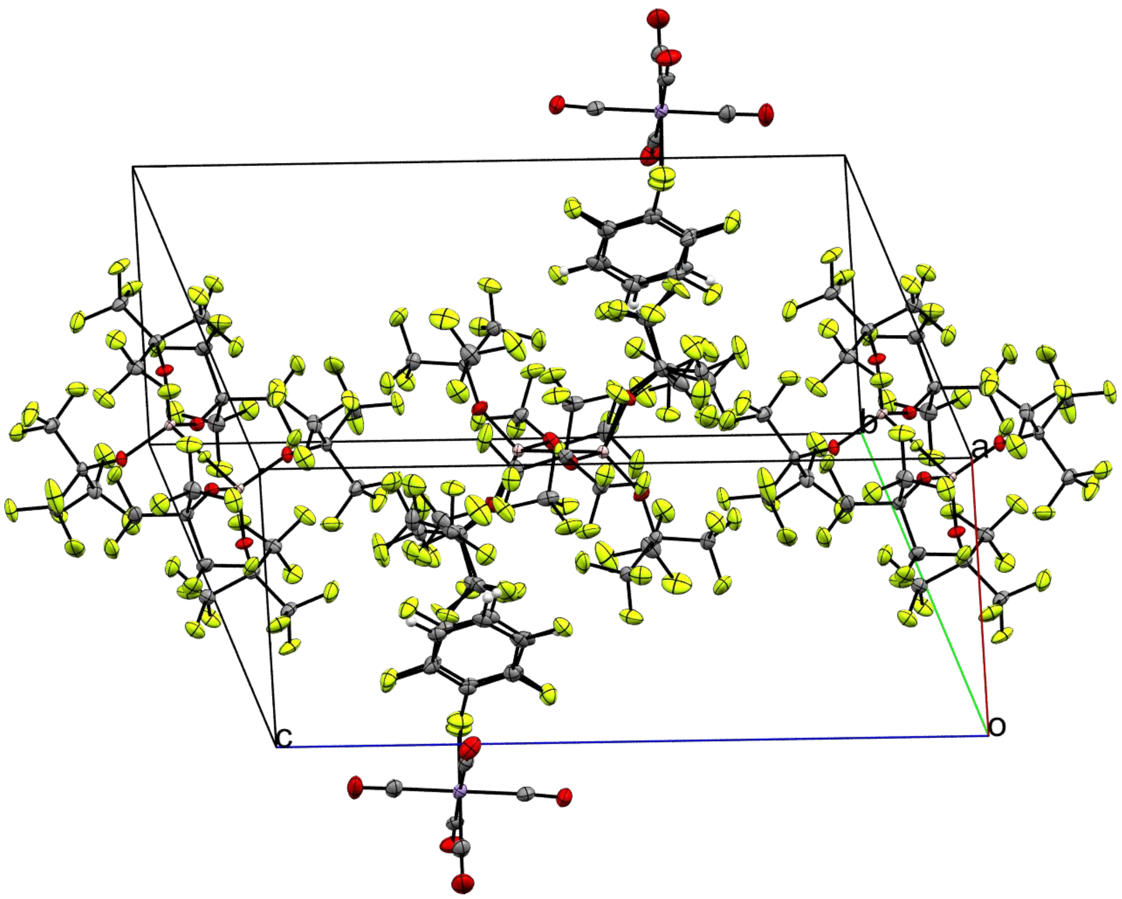

Figure S3: Unit cell of [**2**]$^+$[F{Al(OR^F)$_3$}$_2$]$^-$ with anion and 4FB-disorders. Displacement ellipsoids shown at 50 % probability level. Color code: manganese – lavender, aluminium – rose, fluorine – light green, oxygen – red, carbon – grey, hydrogen – white.

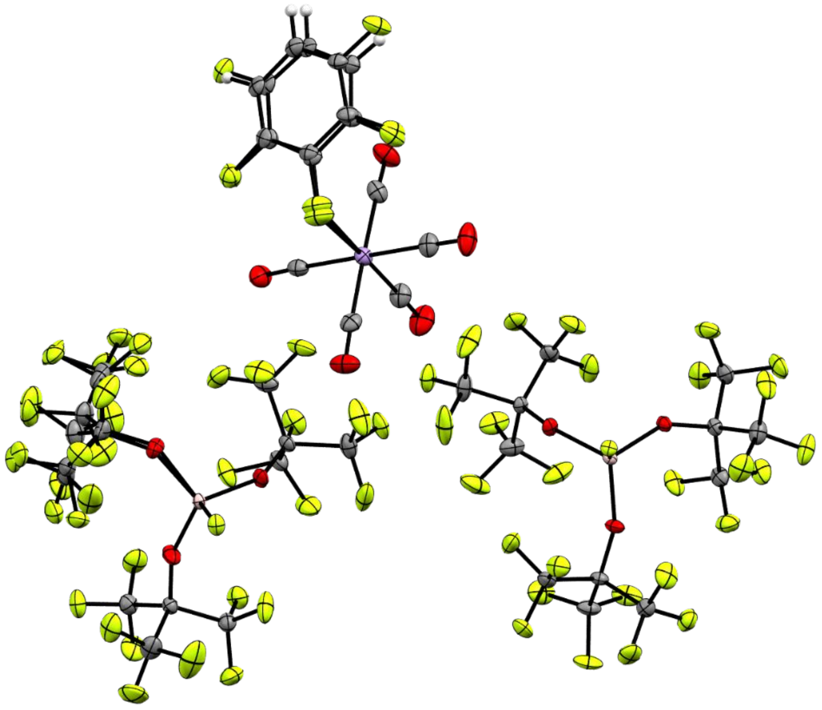

Figure S4: Asymmetric unit of [**2**]$^+$[F{Al(OR^F)$_3$}$_2$]$^-$ with anion and alkane disorders. Displacement ellipsoids shown at 50 % probability level. Color code: manganese – lavender, aluminium – rose, fluorine – light green, oxygen – red, carbon – grey, hydrogen – white.

4. Vibrational Spectroscopy

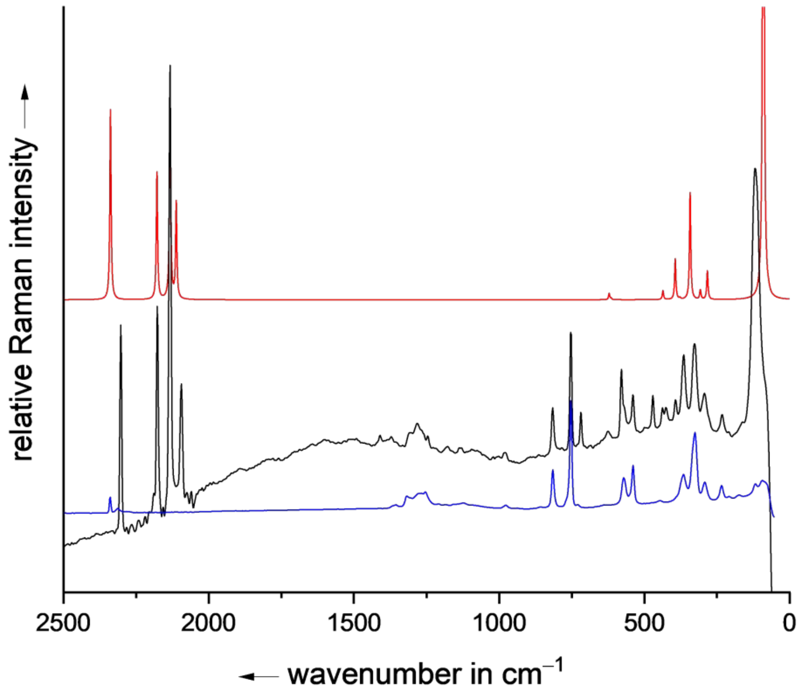

Figure S5: Raman spectrum of crystalline [**1**]$^{+}$[F{Al(OR^{F})$_3$}$_2$]$^{-}$ (black line) in comparison to the Raman spectrum of [NO]$^{+}$[F{Al(OR^{F})$_3$}$_2$]$^{-}$ (blue line), which features only bands of the anion, except the N≡O streching vibration at 2340 cm^{-1} and the DFT-calculated Raman spectrum of [**1**]$^{+}$ (red line) at the B3LYP(D3BJ)/def2-TZVPP level of theory scaled by 0.968 according to Duncan et al.[22]

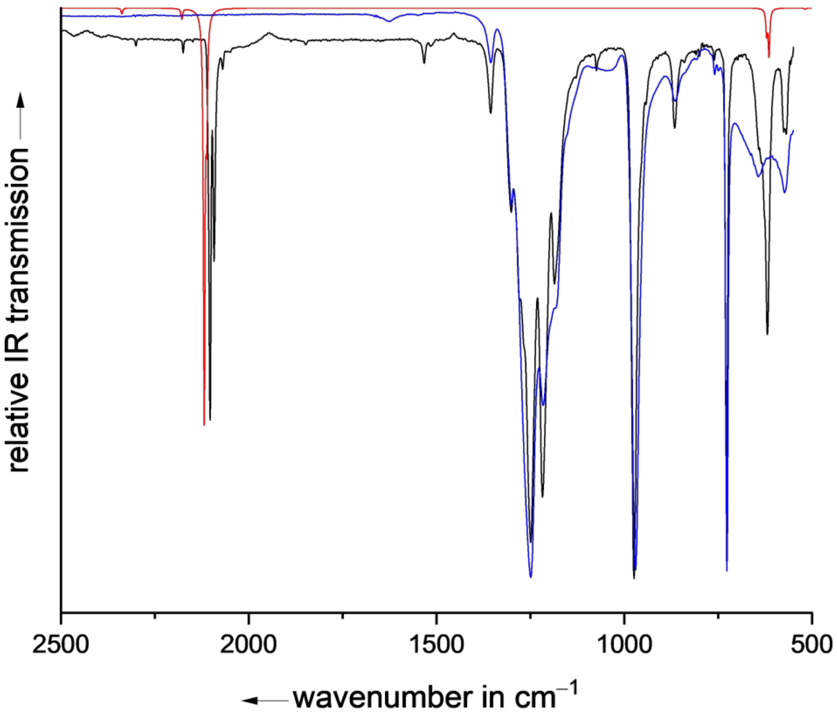

Figure S6: IR spectrum of crystalline [**1**]$^{+}$[F{Al(OR^{F})$_3$}$_2$]$^{-}$ (black line) in comparison to the IR spectrum of [NO]$^{+}$[F{Al(OR^{F})$_3$}$_2$]$^{-}$ (blue line), which features only bands of the anion and the DFT-calculated IR spectrum of [**1**]$^{+}$ (red line) on the B3LYP(D3BJ)/def2-TZVPP level of theory scaled by 0.968 according to Duncan et al.[22]

Table S2: CO-stretching frequencies of different complexes containing the $[Mn(CO)_5]^+$ moiety.

	ν(CO), cm^{-1}			
$Mn^I(CO)_5L$ / Sym.	A_1	B_2	E	A_1
N_2	2177[a]	2133[a]	2103	2094[a]
n-pentane[32]	2166[a]	2123[a]	-	2074[a]
SO_2[33]	2167	-	2061	2040
$[AsF_6]^-$[33]	2167	-	2075	2040
$[OTeF_5]^-$[34]	2155	-	2070	2016
Br^-[35]	2138	-	2052	2007

[a] from Raman spectroscopy

5. NMR Spectroscopy

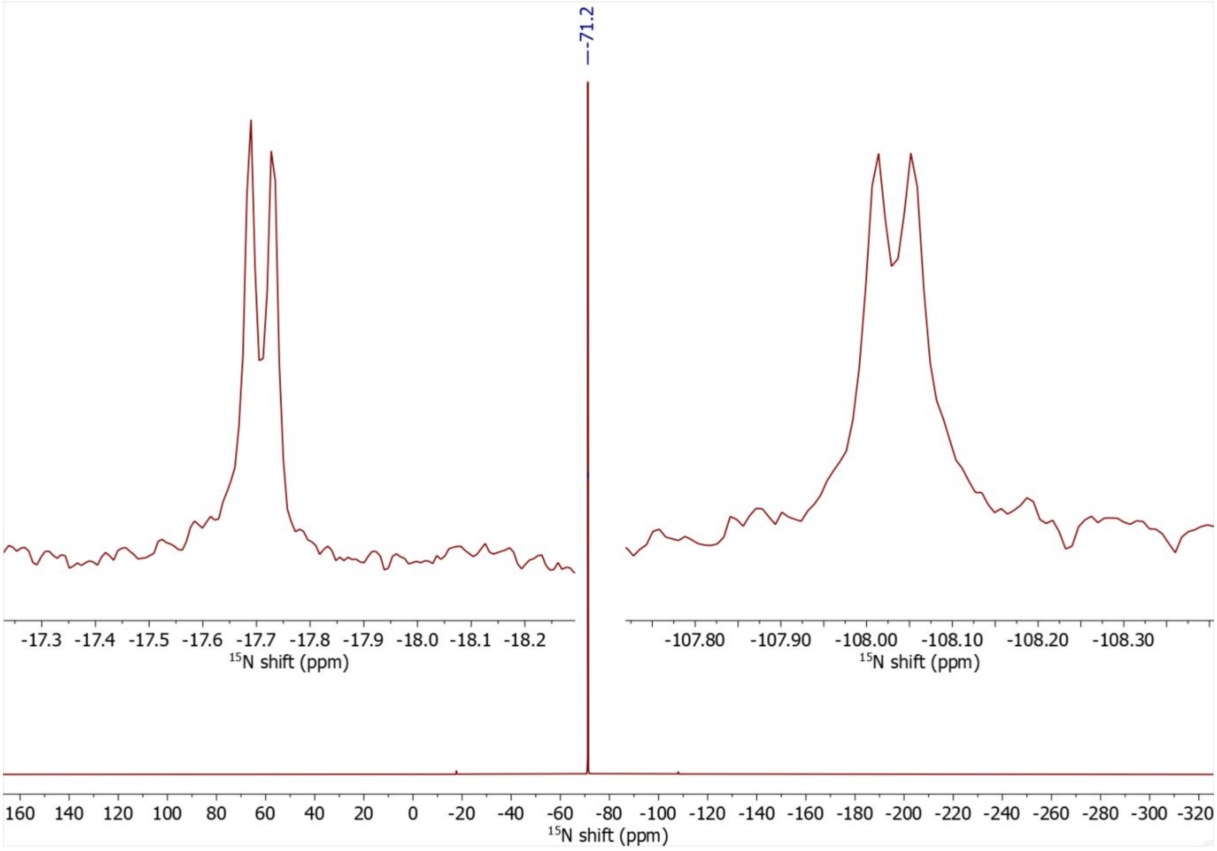

Figure S7: ^{15}N NMR spectrum of *in-situ* generated and isotopically labelled [**1**]$^{+}$[F{Al(OR^{F})$_3$}$_2$]$^{-}$ in 1,1,1,3,3,3-hexafluoropropane under a $^{15}N_2$ atmosphere.

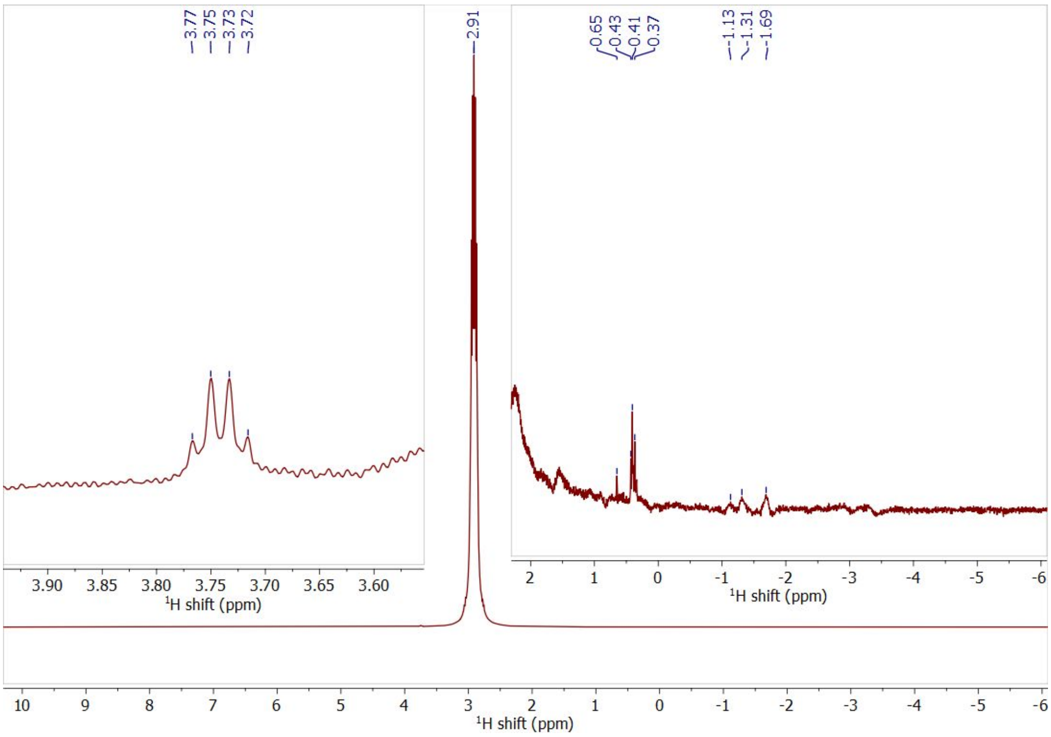

Figure S8: ^{1}H NMR spectrum of *in-situ* generated and isotopically labelled [**1**]$^{+}$[F{Al(OR^{F})$_3$}$_2$]$^{-}$ in 1,1,1,3,3,3-hexafluoropropane in a $^{15}N_2$ atmosphere.

6. DFT Calculations

6.1 AIM Analysis

Table S3: Properties of the bond critical points (BCPs) in the B3LYP(D3BJ)/def2-TZVPP optimized structure of [**1**]$^+$.

#BCP	Atoms	ρ (r$_c$) in e^- Å^{-3}	$\nabla^2\rho$ (r$_c$) in e^- Å^{-5}
BCP1	mn1 - c2	0.726969895	12.2883414
BCP2	mn1 - c3	0.726969895	12.2883414
BCP3	mn1 - c4	0.726969895	12.2883414
BCP4	mn1 - c5	0.726969895	12.2883414
BCP5	c2 - o6	3.453976225	19.2695254
BCP6	c5 - o7	3.453976225	19.2695254
BCP7	c4 - o8	3.453976225	19.2695254
BCP8	c3 - o9	3.453976225	19.2695254
BCP9	mn1 - c10	0.794651366	13.4191504
BCP10	c10 - o11	3.434883637	18.4770668
BCP11	mn1 - n12	0.484309033	12.2271345
BCP12	n12 - n13	4.753473985	-65.268033

Table S4: Calculated QTAIM charges (B3LYP(D3BJ)/def2-TZVPP) in [**1**]$^+$.

Atom	Charge	Volume [Å^3]
Mn	1.093591	8.76648865
C (*cis* to N_2)	1.087424	10.4594434
C (*cis* to N_2)	1.087424	10.4594434
C (*cis* to N_2)	1.087424	10.4594434
C (*cis* to N_2)	1.087424	10.4594434
O (*cis* to N_2)	-1.101805	19.1941883
O (*cis* to N_2)	-1.101806	19.1941883
O (*cis* to N_2)	-1.101806	19.1941883
O (*cis* to N_2)	-1.101806	19.1941883
C (*trans* to N_2)	1.079299	10.1259849
O (*trans* to N_2)	-1.110839	19.2587143
metal-bound N	-0.217829	13.7938805
terminal N	0.213304	16.7521384

6.2 NMR Calculations

Calculated chemical shift = shielding of nitromethane ^{15}N atom – shielding of relevant ^{15}N atom.

Table S5: Comparison of the calculated (PBE0/QZ4P-J) chemical shifts of the ^{15}N atoms in [**1**]$^+$ with the experimental values.

	absolute shielding σ	$\delta_{calc.}$ vs. $\delta_{calc.}(MeNO_2)$	$\delta_{exp.}$	difference ($\delta_{exp.} - \delta_{calc.}$)
$MeNO_2$	–154.4	-	-	-
metal-bound	–49.0	–105.5	–108.0	–2.5
terminal	–151.2	–3.3	–17.7	–14.4
free $^{15}N_2$	–86.3	–68.2	–71.2	–3.1

6.3 Dispersion Interaction Density (DID) Plots

Dispersion Interaction Density (DID) plots illustrate which parts of the fragments contribute most to the dispersion component of the interaction energy (*cf.* Table 1 in in the manuscript). All plots are outputs from the LED calculation, conducted at the DLPNO-CCSD(T1)/def2-QZVPP/ level of theory. Plots were produced using ChimeraX (isovalue = 0.12 kJ/mol/bohr^3 for all complexes).

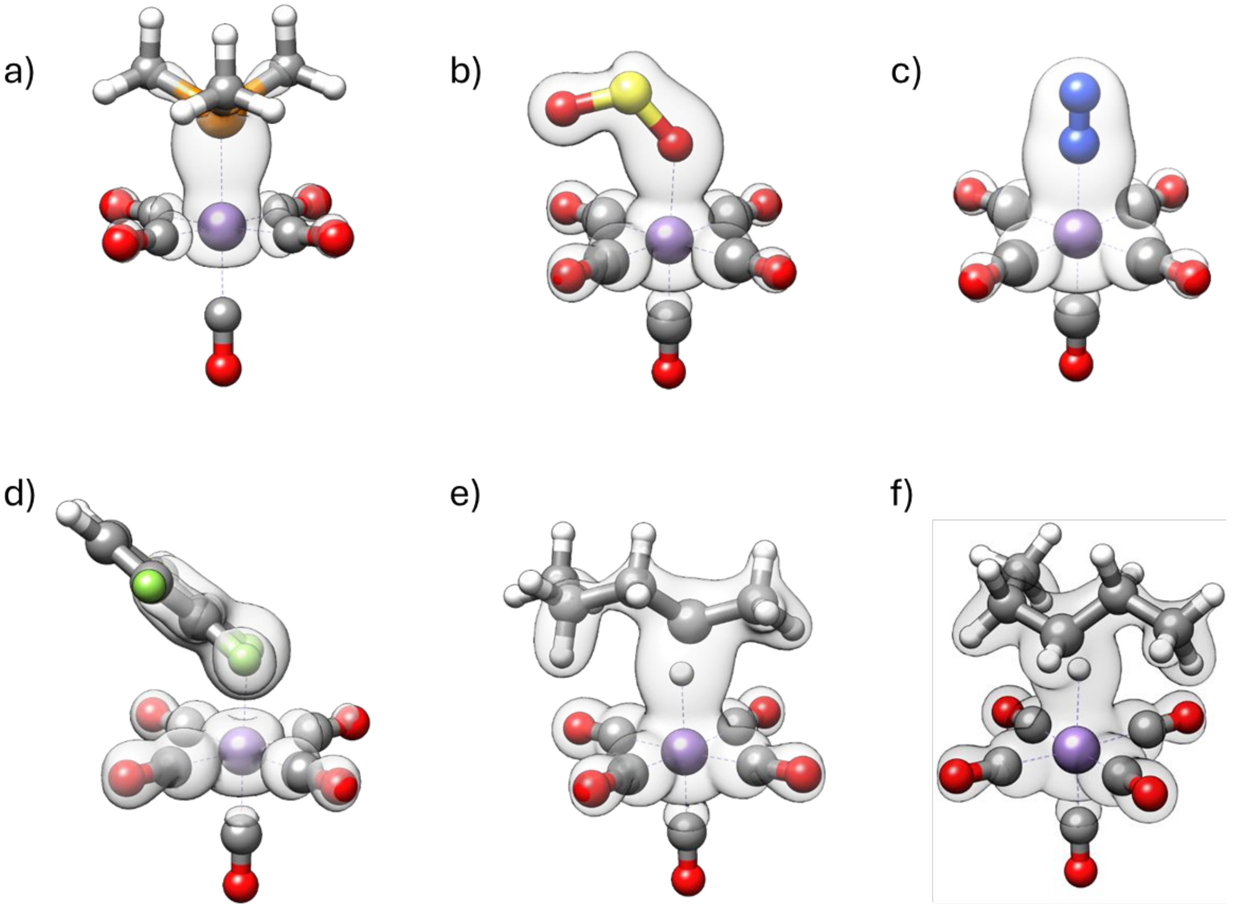

6.4 DFT Optimized Structures

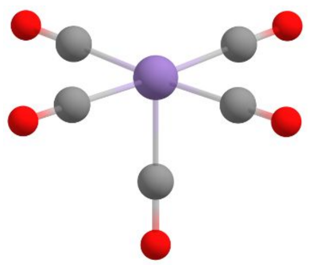

Method: B3LYP(D3BJ)/def2-TZVPP
Symmetry: C_{4v}
Coordinatively unsaturated Mn carbonyl complex
$[Mn(CO)_5]^+$
Energy minimum: –1717.385319 a.u.

Mn	7.06393E-06	7.12704E-06	-0.322249222
C	1.0981E-05	1.924621566	-0.379636377
O	-3.63094E-05	3.048492863	-0.40441292
C	1.924621543	1.11385E-05	-0.379635826
O	3.048492858	-3.62084E-05	-0.404412036
C	-1.924603978	1.11307E-05	-0.379639046
O	-3.048475363	-3.61958E-05	-0.404417229
C	4.92106E-06	4.6985E-06	1.527482856
O	3.59502E-06	2.99631E-06	2.655676644
C	1.10059E-05	-1.924603866	-0.379639247
O	-3.63175E-05	-3.048475249	-0.404417598

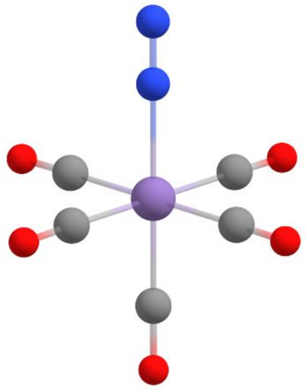

Method: B3LYP(D3BJ)/def2-TZVPP
Symmetry: C_{4v}
Mn dinitrogen complex
$[Mn(CO)_5N_2]^+$
Energy minimum: –1826.940412 a.u.

O	-1.829910407	2.43391875	0.020736126
C	-1.154010037	1.535206612	0.001920818
Mn	-4.35762E-08	-1.41653E-09	-0.00054795
C	-1.39866E-07	-3.24151E-08	1.881334079
O	-1.96829E-07	-7.1059E-08	3.008213762
C	-1.535206529	-1.154010219	0.00192083
O	-2.433918576	-1.829910737	0.020736135
C	1.535206484	1.154010171	0.001920811
O	2.433918544	1.829910672	0.020736075
C	1.154010214	-1.535206418	0.001920738
O	1.829910776	-2.433918453	0.020735929
N	-5.28017E-08	-9.24384E-08	-2.01113399
N	-3.69396E-08	-1.78707E-07	-3.104193362

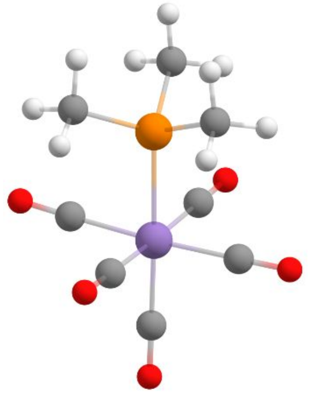

Method: B3LYP(D3BJ)/def2-TZVPP
Symmetry: C_s
Mn trimethylphosphine complex
$[Mn(CO)_5PMe_3]^+$
Energy minimum: –2178.540076 a.u.

Mn	-0.663518677	0.005588857	2.82132E-05
C	-0.546692382	-1.311131803	-1.338896445
O	-0.455103896	-2.094774753	-2.149070219
C	-0.67845874	1.344580735	-1.324782953
O	-0.677166751	2.15236471	-2.115369488
C	-0.546661993	-1.311051514	1.339027174
O	-0.455054307	-2.09464583	2.149245873
C	-2.535767332	-0.085962769	7.02275E-05
O	-3.66401429	-0.146307501	-0.000250224
C	-0.678428949	1.34466783	1.324749907
O	-0.677120081	2.152503823	2.115283292
P	1.726342528	0.049912882	4.4167E-06
C	2.483550603	-1.606718868	-5.23733E-05
C	2.469909436	0.878212702	1.439901171
H	2.182885697	1.928172286	1.464513372
H	3.556301156	0.812120273	1.381780493
H	2.137381868	0.403233334	2.362045405
H	2.1817472	-2.164075804	-0.885006286
H	2.181752584	-2.164136687	0.884865269
H	3.569376045	-1.512114468	-5.17245E-05
C	2.469881589	0.878304285	-1.439854533
H	2.137341341	0.403374559	-2.36201983
H	3.556274829	0.812222248	-1.381758493
H	2.182842525	1.928261472	-1.464402244

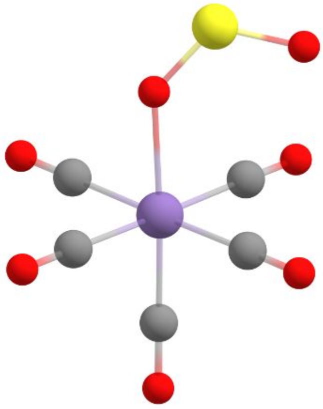

Method: B3LYP(D3BJ)/def2-TZVPP
Symmetry: C_s
Mn sulfur dioxide complex
$[Mn(CO)_5SO_2]^+$
Energy minimum: –2266.031482 a.u.

Mn	-0.006846804	-0.513485103	3.01375E-05
C	-1.216998883	-1.135102733	1.352902099
O	-1.923972711	-1.507687438	2.145529254
C	1.183065375	0.136901376	1.35056948
O	1.890105801	0.499185547	2.148878128
C	-1.216974947	-1.135132398	-1.352848422
O	-1.923937285	-1.507730551	-2.145479436
C	0.873982298	-2.151460802	1.06285E-05
O	1.409677066	-3.145893649	-0.000197812
C	1.18308733	0.136870685	-1.350503454
O	1.890139408	0.49913982	-2.148808455
S	-0.812522528	2.738617048	-3.05758E-05
O	0.570985749	3.098669575	-3.8047E-05
O	-1.107689869	1.302008621	-1.35263E-05

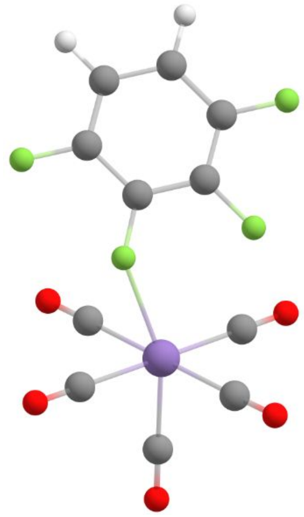

Method: B3LYP(D3BJ)/def2-TZVPP
Symmetry: C_1
Mn tetrafluorobenzene complex
$[Mn(CO)_5(C_6F_4H_2)]^+$
Energy minimum: –2346.60413 a.u.

Mn	0.119779091	-1.335063761	0.000102757
C	-1.45815729	-2.405271358	4.4114E-05
O	-2.392621663	-3.035262591	-1.27355E-07
C	0.133411199	-1.339897144	1.909992799
O	0.166677268	-1.365235881	3.035746989
C	0.133457236	-1.339762189	-1.909782189
O	0.166767535	-1.365071505	-3.035537255
C	1.189546545	-2.844323323	5.54649E-05
O	1.842332362	-3.766198435	2.85572E-05
C	1.644695021	-0.193942298	0.000200124
O	2.550237815	0.476726389	0.000296384
C	-0.71226272	1.705996944	-1.387174019
C	-1.449279742	0.739846131	-0.702393161
H	-2.149132644	0.134001616	-1.258179743
C	-1.449092029	0.739682159	0.702159961
H	-2.148865625	0.133788333	1.257994556
C	-0.712066675	1.705830494	1.386974062
C	0.015096166	2.661291726	0.700481723
C	0.015011729	2.661359324	-0.700649902
F	-0.707430699	1.713626912	2.715671447
F	0.718598334	3.568330275	1.346852637
F	0.71842833	3.568465955	-1.347011763
F	-0.707929546	1.714082227	-2.715873415

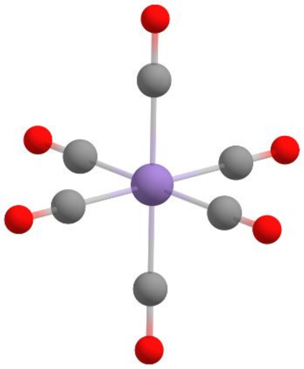

Method: B3LYP(D3BJ)/def2-TZVPP
Symmetry: O_h
Mn hexacarbonyl complex
$[Mn(CO)_6]^+$
Energy minimum: –2266.031482 a.u.

O	-6.8399E-08	-2.70546E-07	3.038990425
C	-5.71371E-09	-1.04688E-07	1.914302509
Mn	7.66399E-08	1.97276E-08	1.07188E-07
C	-7.93272E-08	1.914302443	-8.20443E-08
O	-1.62082E-07	3.038990364	-2.7885E-07
C	-1.914302341	7.92359E-08	4.17407E-08
O	-3.038990261	1.21495E-07	-6.85622E-08
C	1.914302514	4.12301E-08	5.48743E-09
O	3.038990435	5.08329E-08	-1.26882E-07
C	2.88578E-08	-1.87679E-08	-1.914302271
O	-9.02004E-09	-7.51291E-08	-3.038990189
C	-2.84162E-08	-1.91430236	2.20809E-08
O	-9.9549E-08	-3.038990291	-9.4901E-08

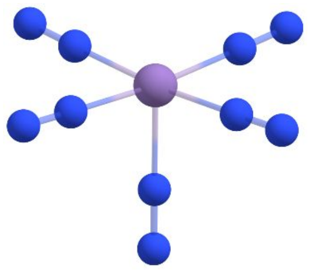

Method: B3LYP(D3BJ)/def2-TZVPP
Symmetry: C_{4v}
Coordinatively unsaturated Mn dinitrogen complex
$[Mn(N_2)_5]^+$
Energy minimum: –1698.32313 a.u.

Mn	-0.000140441	-0.000140463	-0.293450838
N	-4.68356E-05	1.960504408	-0.387881856
N	0.000486924	3.053914816	-0.453544552
N	1.960504415	-4.71346E-05	-0.387882058
N	3.053914811	0.000486464	-0.453544786
N	-1.960781824	-4.71965E-05	-0.387804976
N	-3.054194515	0.000486655	-0.453424209
N	-0.000102268	-0.00010181	1.629507896
N	-8.05235E-05	-7.94353E-05	2.7256558
N	-4.67906E-05	-1.96078181	-0.387805444
N	0.000487047	-3.054194493	-0.453424977

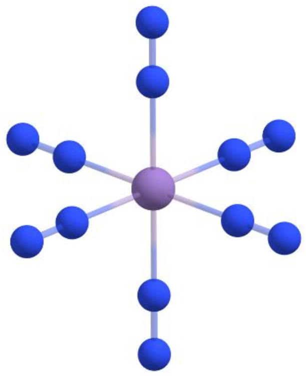

Method: B3LYP(D3BJ)/def2-TZVPP
Symmetry: O_h
Mn hexadinitrogen complex
$[Mn(N_2)_6]^+$
Energy minimum: –1807.877739 a.u.

N	-1.66031E-07	-2.10175E-07	3.066587316
N	-6.94339E-08	-1.1018E-07	1.971798831
Mn	6.64038E-08	-1.03722E-08	-1.11562E-07
N	-6.72746E-08	1.971798944	-3.76578E-08
N	-1.41467E-07	3.066587471	-8.00144E-08
N	-1.971798885	4.24848E-08	6.36549E-08
N	-3.066587398	7.07479E-08	3.21238E-07
N	1.971799079	5.47818E-08	-1.96005E-08
N	3.066587633	1.16127E-07	-1.54647E-08
N	1.0062E-07	4.65431E-08	-1.971799061
N	1.1486E-07	1.35903E-07	-3.066587578
N	-8.11409E-08	-1.971799014	8.30817E-08
N	-1.85837E-07	-3.066587537	2.892E-07

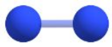

Method: B3LYP(D3BJ)/def2-TZVPP
Symmetry: $D_{\infty h}$
Dinitrogen
N_2
Energy minimum: –109.5218791 a.u.

N	-5.873190405	2.09223657	0
N	-4.782419595	2.11693343	0

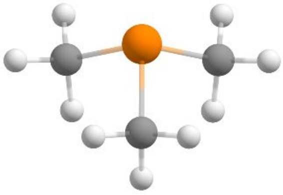

Method: B3LYP(D3BJ)/def2-TZVPP
Symmetry: C_{3v}
Trimethylphosphine
PMe_3
Energy minimum: –461.0468982 a.u.

P	-3.91815E-07	1.90043E-05	-0.601781788
C	-1.409802908	0.813946968	0.278485382
C	1.409800889	0.813949573	0.278484888
C	1.72342E-06	-1.627966133	0.278410032
H	1.465511525	1.863043864	-0.01365485
H	1.30756199	0.755125663	1.364705594
H	2.346153553	0.337383186	-0.013378429
H	-1.46550257	1.863045871	-0.013639866
H	-2.346157127	0.337393326	-0.013393809
H	-1.307573266	0.755107662	1.364706147
H	-0.880823761	-2.200586654	-0.01362518
H	0.880819071	-2.200591955	-0.013639451
H	1.12723E-05	-1.509970375	1.364621329

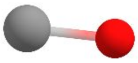

Method: B3LYP(D3BJ)/def2-TZVPP
Symmetry: $C_{\infty v}$
Carbon Monoxide
CO
Energy minimum: –113.3110759 a.u.

C	-5.890199108	2.091851465	0
O	-4.765410892	2.117318535	0

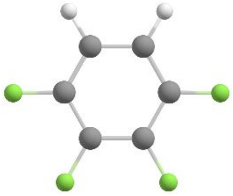

Method: B3LYP(D3BJ)/def2-TZVPP
Symmetry: C_{2v}
Tetrafluorobenzene
4FB
Energy minimum: –629.1838122 a.u.

F	1.356896581	-6.00165E-08	1.840407891
C	0.693914176	1.4306E-08	0.68387311
C	-0.693914175	1.01324E-07	0.683873147
F	-1.356896532	-1.69918E-07	1.840407982
C	-1.37784077	4.80914E-08	-0.525203799
F	-2.718130832	4.06487E-09	-0.50127176
C	-0.69535295	1.32092E-07	-1.726005889
H	-1.25544866	-3.70141E-08	-2.649199697
C	0.695352942	3.34898E-08	-1.726005858
H	1.25544867	-2.46224E-07	-2.649199605
C	1.377840752	1.26053E-07	-0.525203809
F	2.718130797	5.37524E-08	-0.501271712

Summary of the calculated binding energies for the $Mn(CO)_5^+$ complexes

Table S 6: Calculations of relative energies of compounds $[Mn(CO)_5]^+$, $[Mn(CO)_5(L)]^+$ (L = CO, N_2, SO_2, PMe_3, pentane, and 4FB) $[Mn(N_2)_5]^+$ and $[Mn(N_2)_6]^+$.

	Complex[1]	***Energies (a.u.)***[2]	***Binding energies (kJ/mol)***[2]	***Free Energy correction term (a.u.)***[3]	***Free Energies (a.u.)***[4]	***Binding Free energies (kJ/mol)***
**Fragments**	$[Mn(CO)_5]^+$	-1716.184		0.0053	-1716.179	
	$[Mn(N_2)_5]^+$	-1697.140		0.0037	-1697.136	
**Ligands**	PMe_3	-460.485			-460.401	
	CO	-113.188		0.0839	-113.202	
	SO_2	-548.058		-0.0141	-548.075	
	N_2	-109.405		-0.0172	-109.417	
	4FB	-628.507		-0.0128	-628.471	
	Pentane	-197.454		0.0359	-197.323	
**Complexes**	$[Mn(CO)_5(PMe_3)]^+$	-2176.785	**-303.609**	0.1305	-2176.670	**-237.499**
	$[Mn(CO)_6]^+$	-1829.442	**-181.754**		-1829.428	**-122.117**
	$[Mn(CO)_5(SO_2)]^+$	-2264.288	**-121.643**	0.1144	-2264.280	**-69.782**
	$[Mn(CO)_5(N_2)]^+$	-1825.629	**-105.461**	0.0140	-1825.618	**-55.163**
	$[Mn(N_2)_6]^+$	-1806.583	**-100.676**	0.0079	-1806.571	**-47.036**
	$[Mn(CO_5)(4FB)]^+$	-2344.727	**-92.731**	0.0116	-2344.664	**-37.532**
	$[Mn(CO_5)(C2\text{-pentane})]^+$	-1913.676	**-98.005**	0.0113	-1913.519	**-43.814**
	$[Mn(CO_5)(C3\text{-pentane})]^+$	-1913.676	**-99.346**	0.0623	-1913.519	**-44.319**

Notes:

1. Geometries optimized using B3LYP/def2-TZVPP using Orca 6.0.1 with keywords rijcosx, tightscf, verytightopt, defgrid3.
2. Energies calculated using the B3LYP/def2-TZVPP geometries at the DLPNO CCSD(T1)/def2-QZVPP level using Orca 6.0.1 with keywords rijcosx, verytightscf, tightPNO, defgrid3.
3. Free energy correction term includes zero-point energy, enthalpy correction and entropy terms from B3LYP/def2-TZVPP frequency calculation at 298 K using quasi-RRHO method.
4. Using the B3LYP/def2-TZVPP free energy correction term.

7 References

(1) Sellin, M.; Willrett, J.; Röhner, D.; Heizmann, T.; Fischer, J.; Seiler, M.; Holzmann, C.; Engesser, T. A.; Radtke, V.; Krossing, I. Utilizing the Perfluoronaphthalene Radical Cation as a Selective Deelectronator to Access a Variety of Strongly Oxidizing Reactive Cations. *Angew. Chem. Int. Ed.* **2024**, e202406742. DOI: 10.1002/anie.202406742. Published Online: Jun. 6, 2024.
(2) Malinowski, P. J.; Jaroń, T.; Domańska, M.; Slattery, J. M.; Schmitt, M.; Krossing, I. Building blocks for the chemistry of perfluorinated alkoxyaluminates $[Al\{OC(CF_3)_3\}_4]^-$: simplified preparation and characterization of Li^+-Cs^+, Ag^+, NH_4^+, $N_2H_5^+$ and $N_2H_7^+$ salts. *Dalton Trans.* **2020**, *49* (23), 7766–7773. DOI: 10.1039/D0DT00592D. Published Online: Apr. 24, 2020.
(3) Sheldrick, G. M. SHELXT - integrated space-group and crystal-structure determination. *Acta Cryst. A* **2015**, *71* (Pt 1), 3–8. DOI: 10.1107/S2053273314026370. Published Online: Jan. 1, 2015.
(4) Sheldrick, G. M. Crystal structure refinement with SHELXL. *Acta Cryst. C* **2015**, *71* (Pt 1), 3–8. DOI: 10.1107/S2053229614024218. Published Online: Jan. 1, 2015.
(5) Hübschle, C. B.; Sheldrick, G. M.; Dittrich, B. ShelXle: a Qt graphical user interface for SHELXL. *J. Appl. Cryst.* **2011**, *44* (Pt 6), 1281–1284. DOI: 10.1107/S0021889811043202. Published Online: Nov. 12, 2011.
(6) Kratzert, D.; Holstein, J. J.; Krossing, I. DSR: enhanced modelling and refinement of disordered structures with SHELXL. *J. Appl. Cryst.* **2015**, *48* (Pt 3), 933–938. DOI: 10.1107/S1600576715005580. Published Online: Apr. 25, 2015.
(7) Kratzert, D.; Krossing, I. Recent improvements in DSR. *J. Appl. Cryst.* **2018**, *51* (3), 928–934. DOI: 10.1107/S1600576718004508.
(8) D. Kratzert. *FinalCif*. https://www.xs3.uni-freiburg.de/research/finalcif.
(9) Macrae, C. F.; Sovago, I.; Cottrell, S. J.; Galek, P. T. A.; McCabe, P.; Pidcock, E.; Platings, M.; Shields, G. P.; Stevens, J. S.; Towler, M.; Wood, P. A. Mercury 4.0: from visualization to analysis, design and prediction. *J. Appl. Cryst.* **2020**, *53* (Pt 1), 226–235. DOI: 10.1107/S1600576719014092. Published Online: Feb. 1, 2020.
(10) Groom, C. R.; Bruno, I. J.; Lightfoot, M. P.; Ward, S. C. The Cambridge Structural Database. *Acta Cryst. B* **2016**, *72* (Pt 2), 171–179. DOI: 10.1107/S2052520616003954. Published Online: Apr. 1, 2016.
(11) Armbruster, C.; Sellin, M.; Seiler, M.; Würz, T.; Oesten, F.; Schmucker, M.; Sterbak, T.; Fischer, J.; Radtke, V.; Hunger, J.; Krossing, I. Pushing redox potentials to highly positive values using inert fluorobenzenes and weakly coordinating anions. *Nat. Commun.* **2024**, *15* (1), 6721. DOI: 10.1038/s41467-024-50669-3. Published Online: Aug. 7, 2024.
(12) Arnim, M. von; Ahlrichs, R. Performance of parallel TURBOMOLE for density functional calculations. *J. Comput. Chem.* **1998**, *19* (15), 1746–1757. DOI: 10.1002/(SICI)1096-987X(19981130)19:15<1746:AID-JCC7>3.0.CO;2-N.
(13) Treutler, O.; Ahlrichs, R. Efficient molecular numerical integration schemes. *J. Chem. Phys.* **1995**, *102* (1), 346–354. DOI: 10.1063/1.469408.
(14) Becke, A. D. A new mixing of Hartree–Fock and local density-functional theories. *J. Chem. Phys.* **1993**, *98* (2), 1372–1377. DOI: 10.1063/1.464304.
(15) Lee; Yang; Parr. Development of the Colle-Salvetti correlation-energy formula into a functional of the electron density. *Phys. Rev. B Condens. Matter* **1988**, *37* (2), 785–789. DOI: 10.1103/PhysRevB.37.785.
(16) Weigend, F.; Ahlrichs, R. Balanced basis sets of split valence, triple zeta valence and quadruple zeta valence quality for H to Rn: Design and assessment of accuracy. *Phys. Chem. Chem. Phys.* **2005**, *7* (18), 3297–3305. DOI: 10.1039/B508541A. Published Online: Aug. 4, 2005.

(17) Sierka, M.; Hogekamp, A.; Ahlrichs, R. Fast evaluation of the Coulomb potential for electron densities using multipole accelerated resolution of identity approximation. *J. Chem. Phys.* **2003**, *118* (20), 9136–9148. DOI: 10.1063/1.1567253.
(18) Weigend, F. Accurate Coulomb-fitting basis sets for H to Rn. *Phys. Chem. Chem. Phys.* **2006**, *8* (9), 1057–1065. DOI: 10.1039/B515623H. Published Online: Jan. 3, 2006.
(19) Ahlrichs, R. Efficient evaluation of three-center two-electron integrals over Gaussian functions. *Phys. Chem. Chem. Phys.* **2004**, *6* (22), 5119. DOI: 10.1039/B413539C.
(20) Grimme, S.; Ehrlich, S.; Goerigk, L. Effect of the damping function in dispersion corrected density functional theory. *J. Comput. Chem.* **2011**, *32* (7), 1456–1465. DOI: 10.1002/jcc.21759. Published Online: Mar. 1, 2011.
(21) Deglmann, P.; Furche, F.; Ahlrichs, R. An efficient implementation of second analytical derivatives for density functional methods. *Chem. Phys. Lett.* **2002**, *362* (5-6), 511–518. DOI: 10.1016/S0009-2614(02)01084-9.
(22) Assefa, M. K.; Devera, J. L.; Brathwaite, A. D.; Mosley, J. D.; Duncan, M. A. Vibrational scaling factors for transition metal carbonyls. *Chem. Phys. Lett.* **2015**, *640*, 175–179. DOI: 10.1016/j.cplett.2015.10.031.
(23) Lu, T.; Chen, F. Multiwfn: a multifunctional wavefunction analyzer. *J. Comput. Chem.* **2012**, *33* (5), 580–592. DOI: 10.1002/jcc.22885. Published Online: Dec. 8, 2011.
(24) Schmitt, M.; Krossing, I. Terminal end-on coordination of dinitrogen versus isoelectronic CO: A comparison using the charge displacement analysis. *J. Comput. Chem.* **2023**, *44* (3), 149–158. DOI: 10.1002/jcc.26837. Published Online: Mar. 21, 2022.
(25) Becke, A. D. Density-functional exchange-energy approximation with correct asymptotic behavior. *Phys. Rev. A* **1988**, *38* (6), 3098–3100. DOI: 10.1103/PhysRevA.38.3098.
(26) van Lenthe, E.; Baerends, E. J. Optimized Slater-type basis sets for the elements 1–118. *J. Comput. Chem.* **2003**, *24* (9), 1142–1156. DOI: 10.1002/jcc.10255.
(27) van Lenthe, E.; Baerends, E. J.; Snijders, J. G. Relativistic regular two-component Hamiltonians. *J. Chem. Phys.* **1993**, *99* (6), 4597–4610. DOI: 10.1063/1.466059.
(28) van Lenthe, E.; Baerends, E. J.; Snijders, J. G. Relativistic total energy using regular approximations. *J. Chem. Phys.* **1994**, *101* (11), 9783–9792. DOI: 10.1063/1.467943.
(29) van Lenthe, E.; Ehlers, A.; Baerends, E.-J. Geometry optimizations in the zero order regular approximation for relativistic effects. *J. Chem. Phys.* **1999**, *110* (18), 8943–8953. DOI: 10.1063/1.478813.
(30) Guo, Y.; Riplinger, C.; Becker, U.; Liakos, D. G.; Minenkov, Y.; Cavallo, L.; Neese, F. Communication: An improved linear scaling perturbative triples correction for the domain based local pair-natural orbital based singles and doubles coupled cluster method DLPNO-CCSD(T). *J. Chem. Phys.* **2018**, *148* (1), 11101. DOI: 10.1063/1.5011798.
(31) Autschbach, J. Two-component relativistic hybrid density functional computations of nuclear spin-spin coupling tensors using Slater-type basis sets and density-fitting techniques. *J. Chem. Phys.* **2008**, *129* (9), 94105. DOI: 10.1063/1.2969100.
(32) Sellin, M.; Watson, J. D.; Fischer, J.; Ball, G. E.; Field, L. D.; Krossing, I. Promoting Alkane Binding: Crystallization of a Cationic Manganese(I)-Pentane σ-Complex from Solution. *Angew. Chem. Int. Ed.* **2025**, e202507494. DOI: 10.1002/anie.202507494.
(33) Mews, R. Reaction of Transition Metal Carbonyl Halides with $AgAsF_6$ in Liquid SO_2: A Key Reaction for the Preparation of Novel Complex Cations. *Angew. Chem. Int. Ed.* **1975**, *14* (9), 640. DOI: 10.1002/anie.197506401.
(34) Abney, K. D.; Long, K. M.; Anderson, O. P.; Strauss, S. H. Preparation and properties of metal carbonyl teflates, including the structure and reactivity of $Mn(CO)_5(OTeF_5)$. *Inorg. Chem.* **1987**, *26* (16), 2638–2643. DOI: 10.1021/ic00263a017.

(35) Ottesen, D. K.; Gray, H. B.; Jones, L. H.; Goldblatt, M. Potential constants of manganese pentacarbonyl bromide from the vibrational spectra of isotopic species. *Inorg. Chem.* **1973**, *12* (5), 1051–1061. DOI: 10.1021/ic50123a015.
